# Prediction of Snacking Behavior Involving Snacks Having High Levels of Saturated Fats, Salt, or Sugar Using Only Information on Previous Instances of Snacking: Survey- and App-Based Study

**DOI:** 10.2196/57530

**Published:** 2025-04-23

**Authors:** Shaima Dammas, Tillman Weyde, Katy Tapper, Gerasimos Spanakis, Anne Roefs, Emmanuel M Pothos

**Affiliations:** 1 Department of Health Services and Hospitals Administration King AbdulAziz University Jeddah Saudi Arabia; 2 Department of Computer Science City, University of London London United Kingdom; 3 Department of Psychology City, University of London London United Kingdom; 4 Department of Advanced Computing Sciences Maastricht University Maastricht The Netherlands; 5 Faculty of Psychology & Neuroscience Maastricht University Maastricht The Netherlands

**Keywords:** high fats, salt, or sugar snacks, machine learning algorithms, internet data collection, just-in-time interventions

## Abstract

**Background:**

Consuming high amounts of foods or beverages with high levels of saturated fats, salt, or sugar (HFSS) can be harmful for health. Many snacks fall into this category (HFSS snacks). However, the palatability of these snacks means that people can sometimes struggle to reduce their intake. Machine learning algorithms could help in predicting the likely occurrence of HFSS snacking so that just-in-time adaptive interventions can be deployed. However, HFSS snacking data have certain characteristics, such as sparseness and incompleteness, which make snacking prediction a challenge for machine learning approaches. Previous attempts have employed several potential predictor variables and have achieved considerable success. Nevertheless, collecting information from several dimensions requires several potentially burdensome user questionnaires, and thus, this approach may be less acceptable for the general public.

**Objective:**

Our aim was to consider the capacity of standard (unmodified in any way; to tailor to the specific learning problem) machine learning algorithms to predict HFSS snacking based on the following minimal data that can be collected in a mostly automated way: day of the week, time of the day (divided into time bins), and location (divided into work, home, and other).

**Methods:**

A total of 111 participants in the United Kingdom were asked to record HFSS snacking occurrences and the location category over a period of 28 days, and this was considered the UK dataset. Data collection was facilitated by a purpose-specific app (Snack Tracker). Additionally, a similar dataset from the Netherlands was used (Dutch dataset). Both datasets were analyzed using machine learning methods, including random forest regressor, Extreme Gradient Boosting regressor, feed forward neural network, and long short-term memory. We additionally employed 2 baseline statistical models for prediction. In all cases, the prediction problem was the time to the next HFSS snack from the current one, and the evaluation metric was the mean absolute error.

**Results:**

The ability of machine learning methods to predict the time of the next HFSS snack was assessed. The quality of the prediction depended on the dataset, temporal resolution, and machine learning algorithm employed. In some cases, predictions were accurate to as low as 17 minutes on average. In general, machine learning methods outperformed the baseline models, but no machine learning method was clearly better than the others. Feed forward neural network showed a very marginal advantage.

**Conclusions:**

The prediction of HFSS snacking using sparse data is possible with reasonable accuracy. Our findings offer a foundation for further exploring how machine learning methods can be used in health psychology and provide directions for further research.

## Introduction

### General Background

Noncommunicable diseases, such as cardiovascular disease, cancer, and chronic respiratory disease, are currently the biggest threats to health [1]. These are greatly influenced by behaviors, such as poor diet, physical inactivity, and smoking (eg, [2,3]). Modification of the obesogenic environment would be most effective at bringing about change on a large scale [4]. However, such an environmental change is unlikely to occur in a short period. Therefore, most related interventions seek to change people’s responses to their environments. Approaches to achieve these changes have ranged from mass media campaigns at the population level to group-based and individual healthy lifestyle coaching. Over the last decade, digital technologies for supporting a healthy lifestyle have been on the rise, but there is still room for improvement [5-7].

A potential avenue for improving the effectiveness of technology-assisted interventions is providing just-in-time adaptive interventions (JITAIs). JITAIs are designed to predict the points at which a person is likely to be in most need of and most receptive to reminders or assistance for changing a target behavior [8]. For example, a person engaged in an effort to quit smoking could be reminded of their goal at the point at which they are most likely to lapse [9,10]. This may be effective because motivation for a particular behavior can vary over time [11]. Additionally, health-related behaviors may be elicited by cues in the environment (eg, a certain time of the day or walking past a bakery store) and be largely habitual [12-15]. This can make change difficult unless behaviors elicited by these cues are disrupted. Helping a person identify those cues may reduce the undesired behavior in a number of ways, for example, by helping the person avoid the cues, adjust their behavior, or respond to the cues in a different way [15,16]. JITAIs could help people achieve such goals.

There is increasing research into the use of JITAIs (eg, [17,18]). Their development has been greatly boosted by the widespread adoption of powerful personal smartphones. For example, in 2021, 88% of all adults (aged 16 years or older) possessed a smartphone in the United Kingdom [19], with similar statistics throughout Western Europe and North America. Modern smartphones allow increasingly complex data collection from their owners, including date, time, ambient temperature, and location. Furthermore, owing to the high computational capabilities of modern smartphones, many of the required computations (eg, for prediction) can be carried out locally, without the need for distant servers and an active internet connection.

Snacking on foods and drinks that have high saturated fats, salt, or sugar (HFSS) is the focus of this research. Snacking can be defined as “food and beverage intake between meals, including products, such as potato chips, chocolate, and soft beverages” [20]. HFSS foods contribute to poor health [21], and many snacks fall into this category. Indeed, a previous study found that people who are overweight or obese eat an average of 1.3 snacks per day, with 79% of these snacks being high in either fat or sugar [12]. However, reducing HFSS snacking poses many challenges, particularly because it can be triggered by emotional or environmental factors [22]. It can also occur in an automatized (reflexive) way, making it less amenable to conscious control efforts [12-14]. Additionally, snacks that are high in sugar may make a person crave more sugary foods, because consumption of these snacks can lead to a spike and subsequent dip in blood sugar levels [23,24]. Indeed, feelings of hunger and food preoccupation are key reasons cited for snacking [12].

Several sophisticated approaches to predict aspects of maladaptive eating behavior have already been proposed, using ecological momentary assessments (EMAs). For example, Arend et al [25] studied binge eating episodes in clinical participants. The authors reported excellent predictive accuracy based on an EMA protocol with 36 items, including emotional and environmental variables. Based on initial testing, they were subsequently able to identify a smaller subset (n=5-9) of highly valid individualized predictors (EMA items), thereby reducing the need for an extensive EMA protocol. Forman et al [26] similarly investigated the predictive adequacy of a large number of variables concerning dietary lapses. Some questions, such as those relating to cravings or affect, were answered as many as 4 times a day, and others were answered only once. Kaiser et al [27] used data from 2 weeks of EMAs on stress and emotion, together with sensor data, to predict (reasonable accuracy) food cravings. Finally, Spanakis et al [28] tracked several individual states, such as emotions and cravings, which might predict “unhealthy eating events,” including unhealthy snacking and other events, such as consumption of high-calorie food as part of a meal, in people who were overweight or obese. Participants were questioned as many as 10 times a day. Based on the collected data, a bottom-up clustering algorithm was used to arrive at 6 different subgroups of participants characterized by a specific pattern of eating behavior (eg, eating in the evening at home), to enable tailoring the intervention to a specific profile, which was implemented in a randomized controlled trial [29].

Research based on EMAs is valuable because the prediction of a behavior as complex as eating can potentially only be accomplished by considering a multitude of variables, including environmental, psychological, and physiological variables. Moreover, the assessment of these variables takes place in daily life, contributing to ecological validity. However, health interventions based on EMAs typically require long periods to train the machine learning (ML) algorithms for prediction, as well as considerable commitment and motivation from participants. There is therefore interest in exploring whether the prediction of a particular behavior can proceed based on information that is both minimal and easily available, without much effort from participants.

In the domain of mental health, research on so-called digital phenotyping has recently started to develop. Digital phenotyping uses a smartphone as a tool for objective and ecologically valid measurements. This method includes passively obtained data, without needing input from the user. Digital biomarkers, such as sensor technology, geolocation, characteristics of voice and speech, and human-computer interaction, are obtained [30-33]. Through this method, a pattern might be discovered over several weeks (ie, the user is taking too long to respond to messages, is browsing online until late at night, and is mostly at home). This can lead to suspicion that things are not going particularly well for the user, and the suspicion may be increased by the tone, timing, and content of the user’s social media posts. Research has shown that mood states in mood disorders can be predicted using digital biomarkers based on the circadian rhythm [34].

### Prediction of HFSS Snacking Using ML

This research project takes a step in the direction of digital phenotyping for the prediction of unhealthy eating behavior. Specifically, to what extent can HFSS snacking be predicted only based on prior HFSS snacking combined with information that can be automatically or easily collected from a smartphone (date, time, and location)? However, this endeavor might fail due to temporal resolution requirements. If high precision is needed, failure will be inevitable owing to the intrinsic stochasticity of the eating behavior. Another problem is the degree of accuracy that can be achieved after a modest training period, because participants may not have the patience for extended training (eg, Tulu et al [35]), and even with mostly passively collected data, participants still need to indicate instances of HFSS snack consumption.

On the positive side, ML has progressed to such an extent that modern algorithms have many characteristics desirable for the present application, including the capacity to deal with sparse data and efficient learning of time series. For example, as an alternative to recurrent neural networks, which are well suited to time series data, ensemble methods have a good ability to deal with sparse data by reducing the impact of noise and outliers [36]. Therefore, our aim was to compare a selection of ML algorithms, with a view to identify a good algorithm for predicting instances of HFSS snacking based on only prior instances, which were coded in terms of time, day, and location (the latter was encoded in terms of broad categories). The algorithms were chosen to reflect complementary characteristics and be representative of the range of good options currently available. Random forest regressor (RFreg), Extreme Gradient Boosting regressor (XGBreg), feed forward neural network (FFNN), and long short-term memory (LSTM) were considered.

RFreg is a tree-based ensemble method that trains many decision trees simultaneously with bootstrapping followed by aggregation, collectively referred to as bagging. Bootstrapping involves the training of several individual decision trees (between 100 and 300 in the present case) on several subsets of the dataset, using various subsets of available features [36]. Aggregation means that the outputs from the distinct decision trees are combined into a single decision. RFreg is considered to generalize well and be resistant to overfitting, as well as produce high prediction accuracy, because of ensemble learning [37].

XGBreg is another tree-based ensemble method, which uses a form of gradient boosting, relying on the idea that correcting the model’s earlier errors and learning from them can help to improve performance in the future. This is a sequential ensemble learning method where the model tries to improve performance with each iteration [38]. Both RFreg and XGBreg are ensemble learning techniques, but the former builds multiple trees in parallel and then employs an average for prediction, while the latter constructs 1 tree at a time, in a way that is informed from the errors of the previous tree [39].

FFNN is a relatively simple type of artificial neural network, in which information is processed in 1 direction, from input units to units in one or more hidden layers to output units, such that there are no cycles in the connections between the nodes. Hidden layer units apply nonlinear functions to their input, enabling an FFNN to learn complex associations between input and output [40]. An FFNN is trained using gradient descent methods, specifically error backpropagation [41].

Finally, the LSTM model is a kind of recurrent neural network with an architecture designed for learning long-term dependencies in time series [42]. A recurrent neural network includes cycles that feed network activations from earlier time steps as inputs to determine predictions at the present time step. As a result of these recurrent connections, the model creates an implicit recollection of past occurrences, stored in its hidden layer [43]. Recurrent models can process contexts of arbitrary length; the LSTM model has a specific structure designed to store values for longer compared to standard recurrent neural networks. The LSTM model is the only recurrent model that was employed, with the other models operating on a fixed context.

### Summary of the Purpose and Aims

Despite much interest in predicting eating behavior, there has been less work on prediction involving minimal data. Therefore, the feasibility of predicting HFSS snacking using only previous instances of snacking collected across a “practical” length of time (practical in the sense of participant recruitment and engagement) is unclear. Additionally, there is a wide range of ML algorithms. It is of interest to explore the quality of prediction against the assumed characteristics of HFSS snacking behavior, such as sparseness and high noise.

With these considerations in mind, this study aimed to (1) develop an app, which would enable data collection on HFSS snacking with minimum effort from participants; (2) define a sensible problem for characterizing HFSS snacking behavior (prediction of HFSS snacking using previous instances of snacking and limited information [time, location, and day of the week]); (3) apply a range of standard and unmodified ML algorithms to the problem; (4) compare the performance of these algorithms to each other and to some baseline statistical models; and (5) consider whether the task of predicting HFSS snacking from minimal information is feasible and suggest some directions for future work.

## Methods

### Outline of Data Collection

Data on HFSS snacking were collected to examine which ML algorithm was best able to predict such behavior based on only previous instances and minimal information (time, location, and day of the week). We first describe the procedures employed to collect the data.

**Figure 1:**
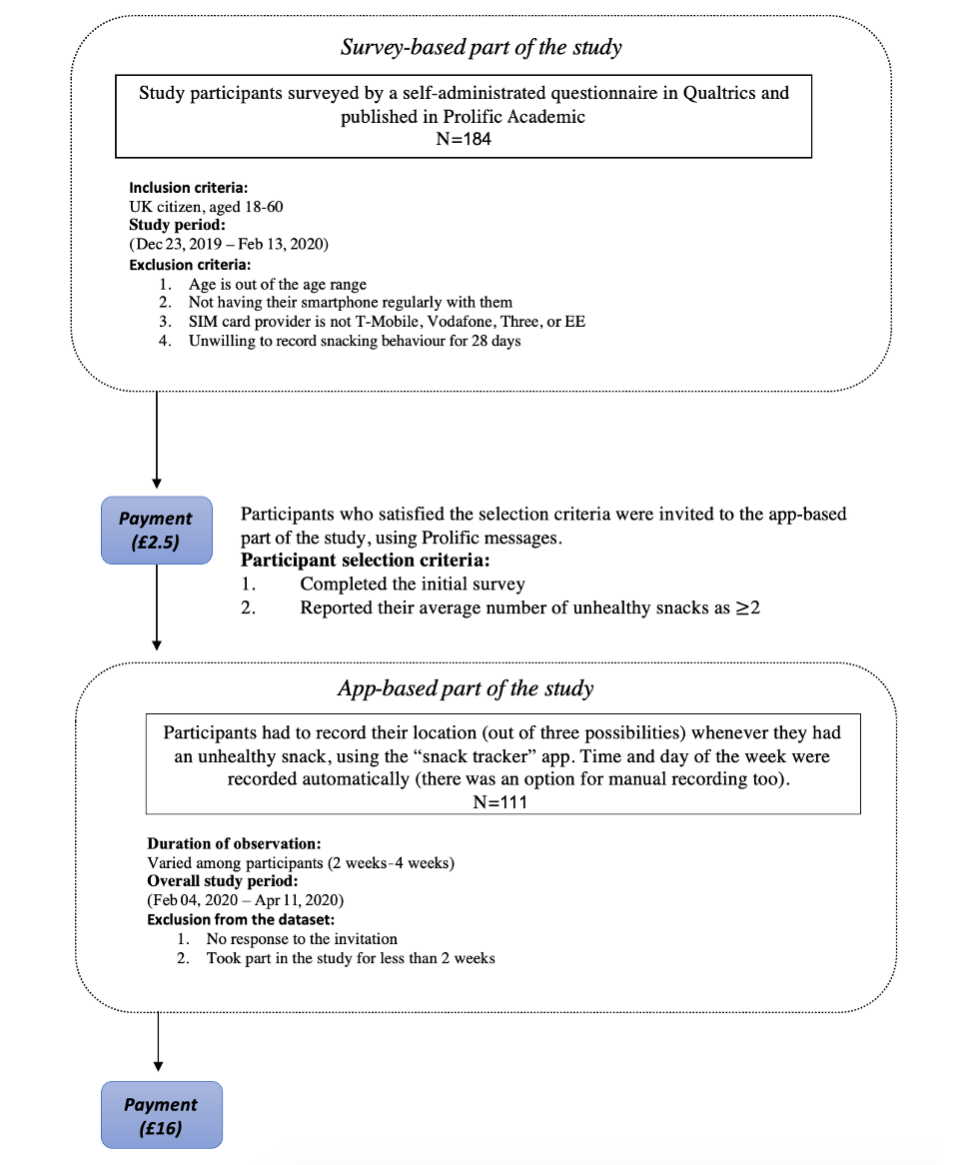
Screening and data collection processes for the UK dataset. Initially, demographic and lifestyle (weight/diet) surveys were used to determine eligibility for the trial. In part 2 (app-based part of the study), participants were asked to record snacking habits for a target period of 4 weeks.

Data were collected in 2 parts. First, a survey was created to explore various assumptions about the target behavior of interest. Second, we implemented an app to obtain data on HFSS snacking. Participants who reported having 2 or more HFFS snacks daily in the first part were invited for the app-based second part of the study. The second part of the study involved monitoring participants’ snacking behaviors for 28 days. The 2 parts of the study are outlined in Figure 1. This dataset is referred to as the “UK dataset.” To increase the ecological validity of our work, we also employed a cleaned version of a similar dataset collected in the Netherlands, which has been described by Spanakis et al [28]. The dataset in the report by Spanakis et al [28] is referred to as the “Dutch dataset.”

The data collection details below concern the UK dataset; corresponding details for the Dutch dataset can be found in the report by Spanakis et al [28].

### Ethical Considerations

For the UK dataset, ethics approval was granted by the Psychology Research Ethics Committee at City, University of London (reference: PSYETH (S/L) 17/18 87). The informed consent form for the first study part (survey part) informed participants that the study was about snacks that are high in sugar, salt, and fat and that the study would be conducted in 2 parts (a brief first part concerning some general questions about eating behavior and a 28-day second part, which would involve participants recording occasions of snacking on their smartphones via an app). The informed consent form for the second part (app-based part) explained in detail about HFSS snacks and informed participants that they would have to record their HFSS snack consumption on an app for 28 days. The informed consent form provided some information about the next steps, if the individual agreed to participate, and the amount of compensation.

All data were collected anonymously. To protect participant anonymity, the Data Protection Office at City, University of London, which is registered with the Information Commissioner’s Office (registration number: Z8947127), was engaged to confirm that any personally identifiable information (PII) was securely collected. Data collection involved 3 companies external to City, University of London: Dev Technosys implemented the app, Linode provided virtual private servers, and Twillo provided programmable messaging services. It was ensured that these companies were compliant with the General Data Protection Regulation (GDPR) and used encrypted communication.

Participants were compensated £2.50 (US $3.13) for their time for the survey part of the study and £16 (US $20.00) for the app-based part.

For the Dutch dataset, ethics approval for the study was provided by the Faculty of Psychology and Neuroscience of Maastricht University in 2013. While the data were not open access, the principal investigators of the study stated availability of the data upon reasonable request [28].

### UK Dataset: Survey-Based Part of the Study

Data for the first part of the study were collected using a self-administered survey designed using Qualtrics [44]. The survey was run on the crowdsourcing platform Prolific Academic [45]. The study was made available on Prolific Academic, and prospective participants decided whether to take part. The study had a target sample of 200 participants on December 23, 2019, and recruitment was closed on February 13, 2020, when this participant number had been reached.

There are no established guidelines for the minimum sample size required for an ML assessment, and a particular ML algorithm can work effectively up to a certain error threshold [46] for a promising but still experimental proposal. Based on previous related research (Spanakis et al [28] recruited 100 participants for an ML study broadly similar to our study), the expected time to train all 4 ML models for each participant, and our budget for paying participants to take part for 28 days, we aimed for approximately 100 participants for the ML assessment. The target of 200 participants for the survey part of the study was an estimate of how many participants would be needed to identify enough participants with a reasonably high intake of HFSS snacks, who would be willing to take part in the app-based part of the study across 4 weeks. The survey part had no inferential value.

Only UK citizens between the ages of 18 and 60 years were allowed to participate in the study. Participants were excluded if they did not have a smartphone or stated that they were unwilling to participate in the follow-up study (ie, the app-based part). Additionally, we only recruited participants having a T-Mobile, O2, Vodafone, Three, or EE mobile phone service, since at the time of running the study, these were the only SIM card providers in the United Kingdom providing services compatible with the Twillo messaging service, which we employed in the second part of the study. After these exclusions, there were 184 participants for the first part of the study.

The survey consisted of questions concerning basic demographics and motivation for healthy eating (Tables S1 and S2 in Multimedia Appendix 1). Specifically, there were 10 questions covering gender, ethnicity, employment status, weight, height, HFSS snacking habit, whether the person is trying to lose weight (2 questions), and whether the person is trying to eat in a healthy way. Participants were not allowed to skip questions. The duration of the survey was about 15 minutes.

### UK Dataset: App-Based Part of the Study – Snack Tracker App

We developed the Snack Tracker app to record unhealthy snacking for this project. The app was designed by SD, and the coding was undertaken by Dev Technosys [47], a company specializing in app development. We created versions of the app for both Android and iOS devices. However, the app is no longer available for use.

**Figure 2:**
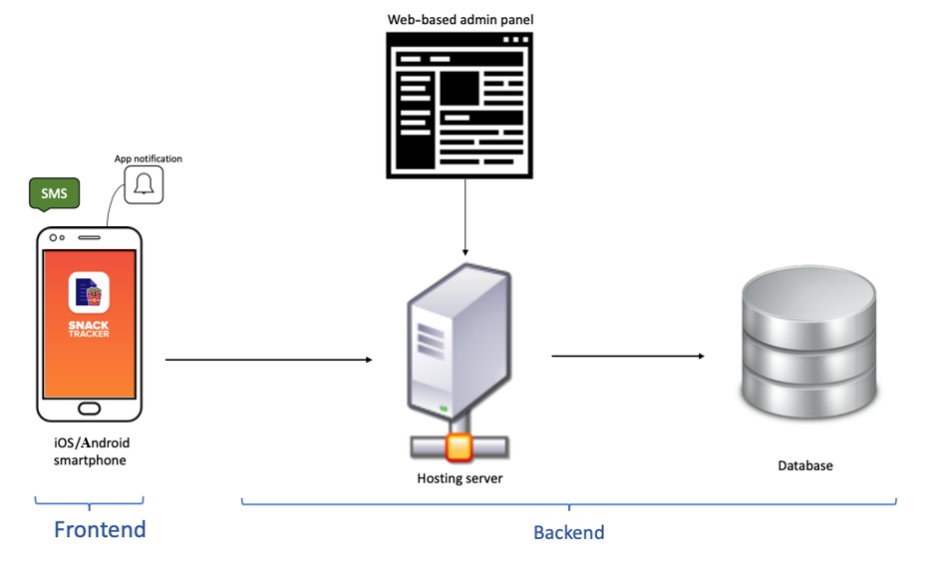
The frontend (user interface) and backend (data storage) of the Snack Tracker app used for participants in the second part of the study to record their intake of snacks with high saturated fats, salt, or sugar over a period of 4 weeks.

**Figure 3:**
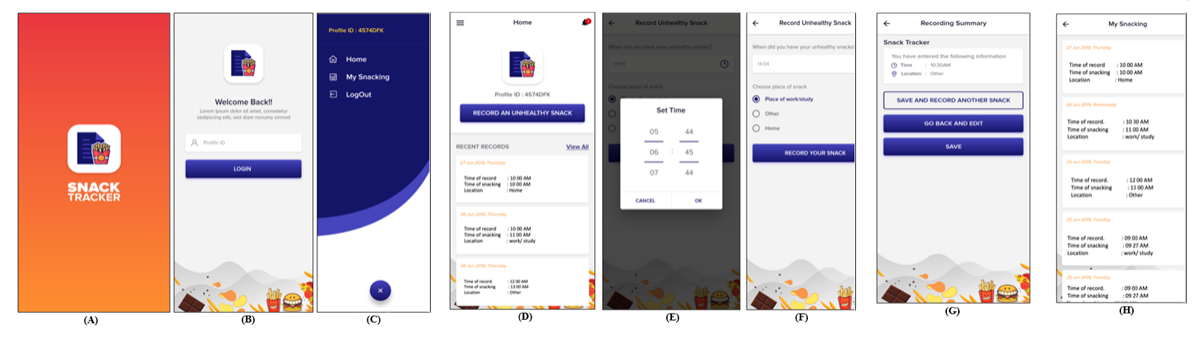
The frontend of the Snack Tracker app: (A) splash screen, (B) login screen, (C) home screen, (D) new snack screen, (E) time recording screen (time picker), (F) location recording screen, (G) recording save screen, and (H) review recording summary.

Mobile app development is usually divided into 2 main components: frontend and backend (Figure 2). Regarding the frontend (the user interface), the app was designed to be easy to use, with a simple sequence of screens (Figure 3 and Figure S1 in Multimedia Appendix 1). The current date and day of the week were automatically captured for each recording to minimize user effort. The app worked online, allowing users to log in and record any snacks they had eaten. In the case of connection loss, the app allowed users to save their records, and the app transmitted the data to the server once the mobile device was connected to the internet.

The app frontend was coded using React Native, which is an open-source JavaScript framework for writing iOS and Android apps. All operations performed by app users and project admins were handled by Rest Application Programming Interfaces (APIs) created in Node JS, which allows running JavaScript on the server side. Rest APIs were used to communicate with the database of participant data (MongoDB) for store and retrieve operations (ie, these APIs acted as a bridge between the app frontend and the database where participant data were stored). The server used in this project was a cloud-based server (Linode), which controlled all operations and allowed the management of the app environment.

The backend part of the app concerned storing the data and user credentials, as well as offering a web-based admin panel to manage the project. Participants accepting the invitation to the app-based part of the study were registered manually using the admin panel to prevent random users from recording data on the app. The backend also handled initial user login and user requests to save and record another snack, go back and edit, or just save an entry (Figure 3).

**Figure 4:**
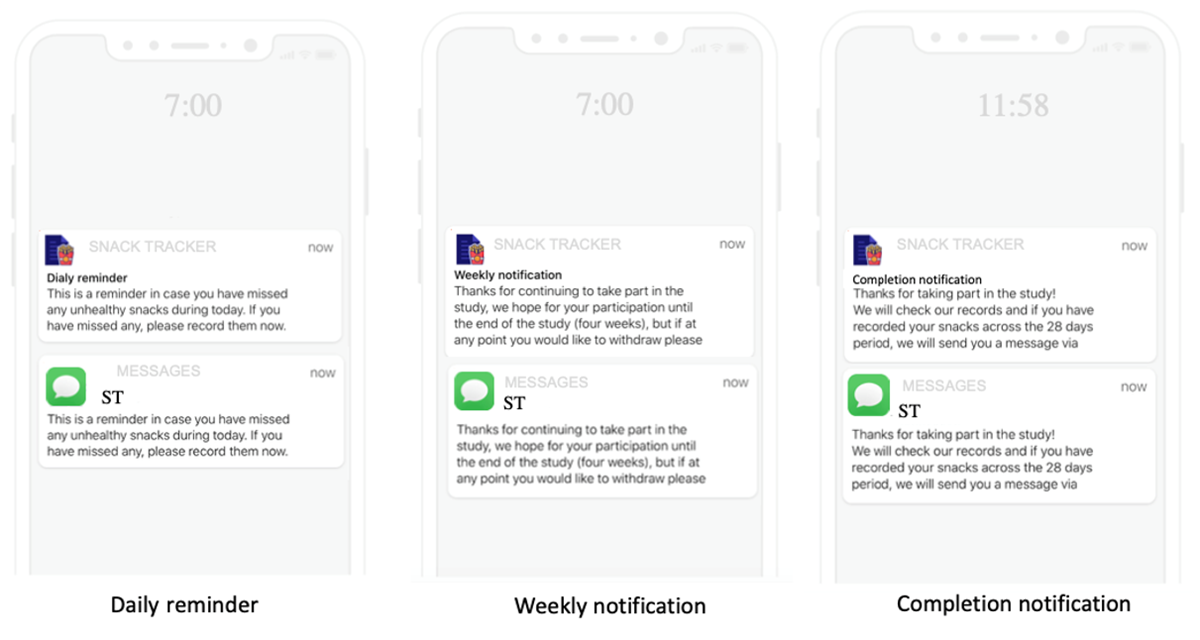
Examples of reminders sent to participants to “nudge” them to record their intake of snacks with high saturated fats, salt, or sugar during the second part of the study (app-based part).

The app development included designing and delivering messages as SMS text messages and app push notifications to keep users engaged and remind them to record snacks (Figure 4). Push notifications could be offered even if participants had no internet access. For automated SMS text messages, Twilio was used, which is a cloud communication tool. The software could programmatically send SMS text messages using Twillo’s web service APIs. For more information, see [48].

### UK Dataset: App-Based Part of the Study – Data Collection

Among the 184 participants who completed the survey-based part of the study, participants were further excluded if they reported consuming fewer than two HFSS snacks per day, leaving 170 participants. We initially invited 100 of these participants to participate in the app-based part of the study, with the invitations sent in 3 stages between February and March 2020. Initially, only 68 participants accepted the invitation, and in the second and third invitation stages, 45 and 25 participants, respectively, were invited, with 27 and 16, respectively, accepting the invitation, resulting in an overall sample of 111.

Participants were instructed to participate for 28 days. However, exact start and end dates of the study differed between participants, as was expected.

During the study, participants were messaged via Prolific if they made only 1 recording or no recordings on any day and if they had made multiple recordings (two or more) each day for a period but then abruptly made a reduced number of recordings. These messages were sent in part to ensure that there were no technical problems with the Snack Tracker app. Additionally, a few Prolific messages were sent to randomly selected participants to check that the reminders regarding snack recording (Figure 4) were received as intended. Informal feedback from participants throughout the study did not indicate any technical problems.

For the purpose of the study, an HFSS snack was defined as any food eaten between the main meals, which was high in either saturated fat, salt, or sugar. Specifically, participants were informed that we are interested in “snacks high in sugar, salt, or fat, which includes… sugary snacks… salty and fatty snacks.” For each of these categories, several examples common in the United Kingdom were provided (eg, biscuits, cake, chocolate, crisps, salted nuts, and salted popcorn). Multimedia Appendix 1 shows exactly how HFSS snacks were explained to the participants. Considering the instructions and several examples of typical items provided, it is likely that participants did not have difficulty with the definition of HFSS snacks. There was no poststudy feedback of any such difficulties. In addition, in keeping with the broad aim of the study (to help people manage their own behavior), there were no strict criteria on what participants should or should not define as an HFSS snack. It was not considered that the app needed to be independently validated, which is in line with similar work, including the study by Spanakis et al [28].

Whenever participants had an HFSS snack, they were asked to record it in the Snack Tracker app. In this mode, they only had to mention the location (coded as home, place of work, and other; although a GPS-based method would aid in this process, this method was not possible in our app) as the time and day of the week were saved automatically. If participants had 2 HFSS snacks at the same time, this would be recorded as a single snacking instance. Throughout the study, participants received 3 kinds of notifications (Figure 4). Daily reminders were sent at 7 PM, asking participants to record any instances of HFSS snacking that they missed. In this mode of the Snack Tracker app, participants had to manually indicate the time and day of the week as well as the location. Additionally, at the end of each week, participants were sent a notification (instead of the daily notification) to keep them engaged and provide information or ask questions (eg, to ask about any technical problems; Multimedia Appendix 1). The final notification was sent at the end of the 28-day period (from the first app recording) to instruct the participants that the study had ended, thank them for their participation, and offer them a completion code for Prolific Academic.

### Dutch Dataset: Brief Notes

The Dutch dataset was collected by a research group at the Faculty of Psychology and Neuroscience at Maastricht University (Study I in the report by Spanakis et al [28]). The dataset was collected with an app called Think Slim. The sample consisted of 57 participants who were overweight and 43 participants with a healthy weight in the Netherlands. This study employed EMAs. Data on 15 variables were collected (eg, mood, activity, and location), and the variables were monitored across 8 to 10 measurements per day depending on the participants’ waking and sleeping times. Spanakis et al [28] aimed to study eating behavior in general (including main meals and snacks, both unhealthy and otherwise), with a view to identify participant clusters and provide adaptive feedback for improving eating behavior. Accordingly, we extracted measurements corresponding to HFSS snacks from the general eating behavior data [28] to create the Dutch dataset. Only “eating moments” were used from the original dataset [28]. Eating moments were event contingent (ie, they were initiated by users whenever they were about to eat something). Therefore, users were not prompted or reminded to record their eating behavior.

The Dutch dataset was already present when the data collection for the UK dataset was planned, and there is a reasonable question as to why we did not adopt a sampling approach similar to that used for the Dutch dataset. In brief, the purpose of the UK dataset was different from that of the Dutch dataset. For the UK dataset, the priority was to collect snacking information in a way that was as nonintrusive as possible. Moreover, we wanted to focus on individuals with some snacking behavior in the first place, since sparsity of data would complicate the application of ML. On the other hand, the purpose of the Dutch dataset was to explore prediction using a range of variables, with a focus on eating behavior in general and not just snacking, and it aimed to understand differences in daily lifestyle between people with a healthy weight and people who are overweight. Accordingly, the extent of snacking behavior was less relevant.

### Data Preprocessing

Regarding the UK dataset, it was decided to exclude participants who did not remain in the study for its intended duration.

Some basic operations were carried out to remove erroneous entries and ensure consistency between the UK and Dutch datasets. In the UK dataset, we removed missing and duplicate entries and ensured that measurement units for height and weight were the same across participants. In the Dutch dataset, we translated data from Dutch to English and manually reclassified 950 locations, originally in free text, into the 3 categories employed in the UK dataset. Finally, HFSS snacks were extracted from the general information about meals or snacks. This involved focusing on data concerning food items, such as burgers, chocolate bars, strawberries, and pasta, and identifying the items considered as snacks. Each snack was manually categorized as healthy or unhealthy (HFSS).

Data were coded in terms of the following 3 features: location (home, place of work, and other), day of the week (Monday to Sunday), and time. A time bin feature was constructed, and there were 4 large and 12 small time bins. A regular day was divided into 4 or 12 time bins. In the former case, the time bins were early morning (midnight to 05:59 AM), morning (6:00 AM to 11:59 AM), afternoon (noon to 4:59 PM), and evening (5:00 PM to 11:59 PM). In the latter case, the first time bin started at midnight and had a 2-hour duration, and every subsequent time bin had a 2-hour duration, resulting in a total of 12 time bins. Models were trained and evaluated for these 2 encodings of the time variable.

As is common in ML, for the 2 nominal variables in the datasets (location and day of the week), one-hot encoding was used, converting each variable to separate variables that take the value 1 or 0 to indicate the presence or absence of different levels of the variable [49]. The time bin variable was kept in its numerical format.

Finally, although additional data were collected from participants in the United Kingdom, including motivation for healthy eating, these additional variables were not considered in this study for practical reasons and for meeting the focus of snacking prediction using minimal information.

### Computational Methods

We employed 3 fixed context models (RFreg, XGBreg, and FFNN) and 1 recurrent model (LSTM). Fixed context models require a fixed input size, which can be achieved by windowing a longer sequence. Windowing is a technique used to divide a longer sequence into smaller, fixed-length sequences. Due to the sparseness of our data, we decided to treat each individual data point as a separate window for prediction, rather than grouping them into larger sequences. This approach is often used when the data are not abundant enough to create longer sequences, and it can simplify the modeling process for the algorithms. Recurrent models can process an input sequence of arbitrary length. They function in cycles, during which the activation from the previous time step is used as input (together with other information) for the current time step. For the single recurrent model in the study, we used the observation sequences of 4 time steps. In our study, the day was divided into different time bins, including a case of 4 time bins (early morning, morning, afternoon, and evening). While the 4 time steps in this model do not directly correspond to the day time divisions, they broadly align with it, and this was sufficient for our analysis.

Some models can benefit from standardization, normalization, and dropout regularization more than others, and these techniques were explored accordingly. Feature scaling (such as standardization or normalization) was considered for the FFNN and LSTM models. Neural networks often benefit from having consistent scales across features. This is because neural networks learn complex relationships and patterns among features, which can be influenced by the differing scales of features, if not appropriately managed [50,51]. On the other hand, tree-based models, such as RFreg and XGBreg, operate by splitting nodes based on feature thresholds and thus are less sensitive to feature scaling. For the FFNN model, we standardized features using the formula *z_i_* = (*x_i_* – µ) / σ, where µ and σ are the mean and SD values of the variable, respectively; *z_i_* indicates the standardized value; and *x_i_* is the original value. For the LSTM model, we employed the normalization *y_i_* = *x_i_* – min (*X*) / (max (*X*)∙min (*X*)).

Dropout was also explored. It is a regularization technique that randomly sets to zero (drops out) a percentage of the features during training [52]. Dropout regularization introduces randomness during training and prevents overspecialization of units, which can improve generalization. This is important for neural networks, as they can overfit the training data. Accordingly, we evaluated and ended up retaining dropout regularization for the FFNN and LSTM models. Tree-based models use other (inherently incorporated) mechanisms, such as feature selection, bootstrapping, and ensemble aggregation, to manage overfitting.

**Figure 5:**
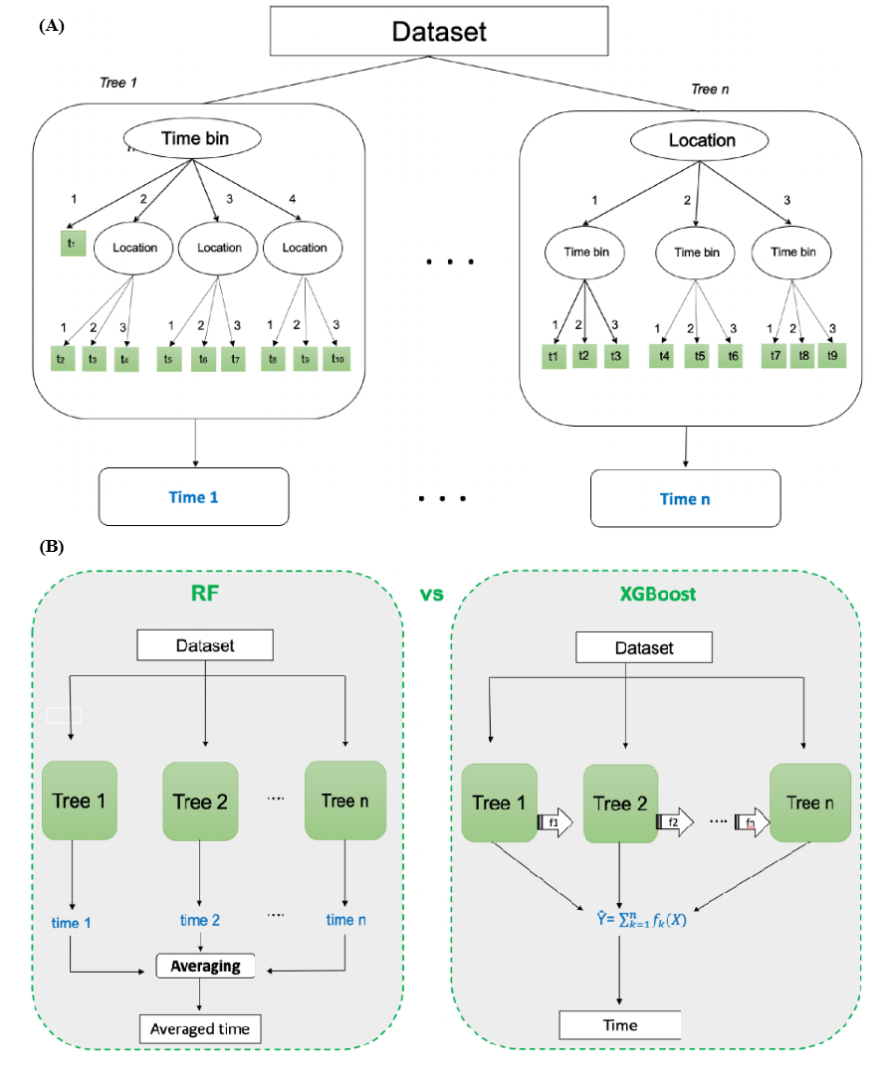
The 2 panels of the figure illustrate the differences between the 2 random forest machine learning algorithms in the study. (A) Both random forest regressor (RFreg) and Extreme Gradient Boosting regressor (XGBreg) are based on the same building block, a decision tree, which is a set of rules built from the dataset. In our case, the aim is to predict the time until the next snack having high saturated fats, salt, or sugar in minutes. n is the number of trees in the model, selected based on cross-validation performance. Time k is the expected time until the final unhealthy snack is predicted using Tree k. (B) For RFreg (on the left), the model creates several different trees and then averages predictions. For XGRreg (on the right), there is a sequence of trees, which progressively refine the prediction. Splits in a decision tree correspond to the data points associated with a node being divided and the parts assigned to child nodes. RF: random forest; XGBoost: Extreme Gradient Boosting.

We executed a total of 5 experiments for each model (5-fold cross-validation) to evaluate their performance. In each experiment, the models were evaluated based on their predefined configurations (Table 1). Both random forest and XGBoost are based on decision trees, and Figure 5 illustrates their differences as well as some of the main parameters.

**Table 1:** Hyperparameters for each of the machine learning algorithms used to analyze the data collected about consumption of snacks with high saturated fats, salt, or sugar during the app-based part of the study.

Model and hyperparameter			Value or range
**RFreg^a^**			
	n_E_, number of estimators (trees in the forest)	150	
	Min_ss,_ minimum number of samples an internal node must cover to consider splitting (when a node has fewer samples than this value, it is regarded as final and called a terminal node or leaf)	2	
	Max_depth_, maximum number of splits that each tree is permitted to execute	Not user specified (nodes are expanded until leaves are pure or contain less than Min_ss_)	
**XGBreg^b^**			
	n_E_, number of boosting rounds (estimators)	100	
	Min_SS_	2	
	Max_depth_	6	
	LR, learning rate (step size for each boosting iteration)	0.3	
	Gamma^c^ (how much the loss must be decreased by a split in order for that split to occur)	0.05	
**FFNN^d^**			
	Number of hidden layers	4	
	Dropout regularization^e^	0.5, after the 2nd and 4th hidden layers	
	Number of neurons at the hidden layer (NHL)	1st hidden layer: 32; 2nd hidden layer: 32; 3rd hidden layer: 8; 4th hidden layer: 8	
	Activation function	ReLU^f^, in the dense and output layers	
**LSTM^g^**			
	Number of neurons in the LSTM layer (NLL)	128	
	Number of hidden layers	3	
	Dropout regularization^e^	0.5, after the 3rd hidden layer	
	NHL	64	
	Activation function	ReLU, in the dense and output layers	

^a^RFreg: random forest regressor.

^b^XGBreg: Extreme Gradient Boosting regressor.

^c^In relation to gamma, loss is the function minimized during model training. It is based on the difference between the current output of the machine learning model and the target (ie, the true value).

^d^FFNN: feed forward neural network.

^e^Dropout regularization is a technique that randomly switches off neurons in a neural network during training to avoid overfitting. The number given refers to the fraction of neurons switched off.

^f^ReLU: rectified linear unit.

^g^LSTM: long short-term memory.

All algorithms had the same objective, which was to predict the time until the next HFSS snack (in minutes), given the current time bin, location (work, home, and other), and day of the week. Specifically, given the presence of an HFSS snack in a time bin, the ML algorithms attempted to predict the number of minutes to the next HFSS snack (snack to snack interval). ML algorithms were applied separately to the snacking data from each participant to take into account individual differences in how the available predictors can be related to snacking behavior. In ML terms, the question is whether there is structure in the data to predict the density of snacking instances, based on the available predictors, location, time bin, and day of the week. To summarize the main approach, we used an input of fixed size, comprising current location, current day of the week, and current time bin, for RFreg, XGBreg, and FFNN. The LSTM model processed data as a sequence of inputs.

To appreciate the capacity of the ML models to extract regularities from the data, 2 baseline models were explored. The first was a simple linear regression model, which attempted to predict snacking instances based on a linear combination of available features, which were suitably weighted. The second was a basic baseline model corresponding to prediction based on the grand mean of snacking (computed separately for each participant) across the time bins.

Five-fold cross-validation was applied on all fixed-context models. In each data split, the datasets were divided into 2 parts (80% for training and 20% for testing). That is, 20% of the data reserved for testing constitutes out-of-sample validation of the models. The error (mean absolute error [MAE]) values were computed on the part of the dataset left for testing (20%). This procedure was repeated 5 times, with each iteration corresponding to a different, randomly determined partition of the data into training and test parts. Reported MAE values were averaged across these 5 iterations. We ensured that data distributions in the training and testing data subsets were similar to those in the whole dataset, using stratified data sampling [53]. Regarding the choice of using 5-fold cross-validation, there is a question of whether it would make sense to have more “folds.” However, each different “fold” increases the number of simulations that must be run and leads to more unbalanced training and testing subsets. Five-fold cross-validation is a fairly standard approach.

For the LSTM, a time series cross-validation was employed. Time series cross-validation is more suitable for temporal data modeling, because the training set only includes the observations occurring before those in the testing set. It begins with a small subset of data for training that is successively extended to generate new predictions [54]. Because of the sparseness of the data, it was decided to use time series cross-validation with only 2 splits.

MAE is the main evaluation metric for the models. MAEs were computed as differences in minutes between the time of the current snack and the predicted time for the next snack. This is because our research focus was the time difference between the prediction and the actual time until the next HFSS snack occurrence. Additionally, residuals allow quantification of positive and negative errors, which were employed in a further analysis (related to the creation of hypothetical interventions). Residuals were calculated as true values minus predicted values. Thus, positive residuals indicate that the predicted time is earlier than the observed time, and negative residuals indicate that the predicted time is later than the observed time. It is important to note that the MAE is computed as the average of the absolute values of the residuals. Residuals were examined as standard, and corresponding plots are provided in Multimedia Appendix 2.

## Results

### Preliminary Notes

Regarding the UK dataset, as expected, some participants did not stay in the study for its intended duration (4 participants). Majority of the participants (107/111) recorded their HFSS snacks for the full 28 days or more. Among these participants, there were 34 female and 73 male participants, with an average age of 32.85 (SD 10.10) years. Some participants continued recording snacking for an additional 6 to 11 days after reaching the 28-day target and after receiving the completion message. These additional data were used in the analyses. Overall, the total number of recordings across all participants was 5391, which reduced to 4978 data points after data cleaning. There were some positive comments about the study in general. For example, participants mentioned that recording their snacks made them aware of the amount consumed daily and helped them reduce food intake.

There were 413 missing and duplicate entries in the UK dataset. Regarding duplicates in the UK dataset, when participants recorded their snacking instances, they might have pressed the submit button twice, which resulted in 2 instances of the same event. However, such duplicate instances were few (approximately 7.7%). In each participant, the total number of recorded snack instances ranged from 7 to 157.

The Dutch dataset included 3705 data points. In each participant, the number of recorded snack instances ranged from 6 to 348. Demographic and other characteristics of the Dutch dataset can be found in the original publication [28].

The sizes of the training and testing sets were 3982 and 996, respectively, in the UK dataset and 2778 and 927, respectively, in the Dutch dataset.

We noted that normalization slightly enhanced the results from the LSTM model but not the FFNN model.

### Model Results

**Figure 6:**
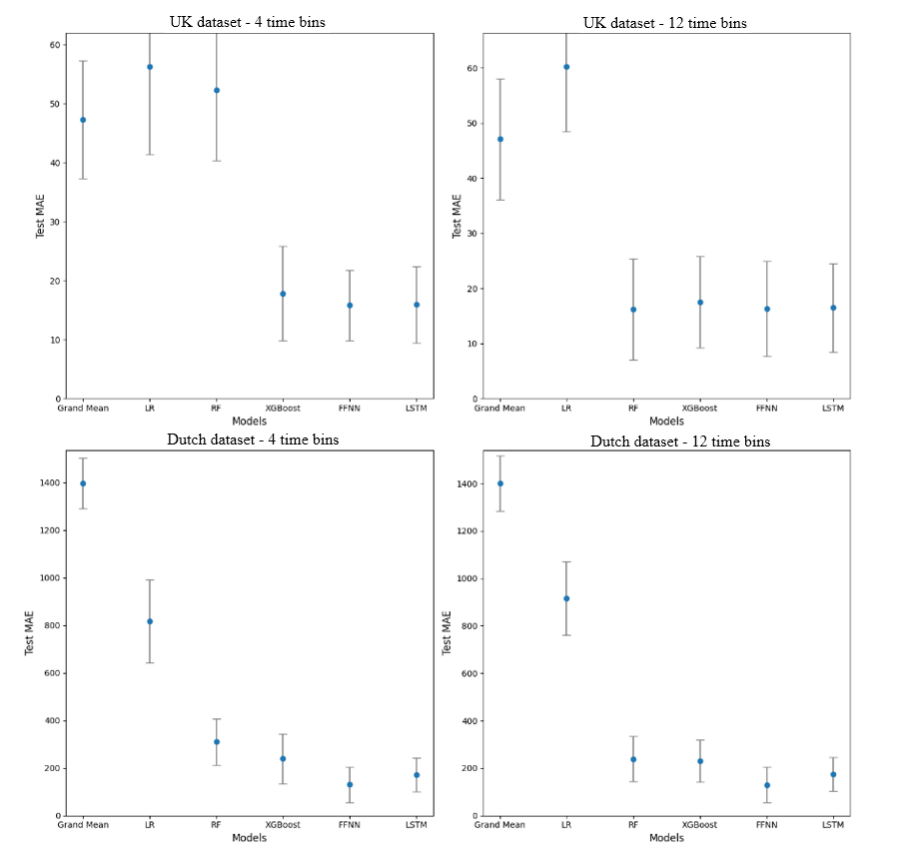
A graphical illustration of the relative performance of the machine learning algorithms (random forest regressor [RFreg], Extreme Gradient Boosting regressor [XGBreg], feed forward neural network [FFNN], and long short-term memory [LSTM]) for predicting consumption of snacks with high saturated fats, salt, or sugar in the UK and Dutch datasets and using 2 different approaches for partitioning time. Performance is quantified using the average mean absolute error (MAE) values from Table 2 (lower values are better). The error bars indicate the IQR. LR: logistic regression; RF: random forest; XGBoost: Extreme Gradient Boosting.

Table 2 and Figure 6 show the MAEs for the 4 models using all input features. In brief, the RFreg model performed very poorly with the UK dataset using 4 time bins. In contrast, the XGBreg, FFNN, and LSTM models all demonstrated reasonable performance with the UK dataset using 4 time bins, achieving lower MAE values and indicating better predictive ability compared to the RFreg and baseline (grand mean) models. When using 12 time bins for the UK dataset, all 4 models performed relatively similarly and demonstrated satisfactory performance. It is not possible to compute significance values from MAE differences in such an ML analysis. This is because to compute statistical significance, some knowledge of the sampling distribution of MAE differences is needed if the null hypothesis (there is no structure in the time series) is true, which is not possible. The best guide for the interpretation of MAE values is the MAE of the baseline (grand mean) model.

**Table 2:** Performance of the machine learning algorithms for predicting consumption of snacks with high saturated fats, salt, or sugar in the UK and Dutch datasets.

Dataset and model		Training set with 4 time bins (MAE^a,b^)	Testing set with 4 time bins (MAE^b^)	Training set with 12 time bins (MAE^b^)	Testing set with 12 time bins (MAE^b^)
**UK dataset**					
	Grand mean	29.2	47.3	29.2	47.1
	LR^c^	9.3	56.3	6.8	60.3
	RFreg^d^	14.7	52.3	6.2	16.2
	XGBreg^e^	4.8	17.8	2.3	17.5
	FFNN^f^	15.3	15.8	15.3	16.3
	LSTM^g^	14.7	15.9	14.8	16.5
**Dutch dataset**					
	Grand mean	1049.0	1397.3	1049.0	1400.1
	LR	116.1	816.1	99.2	914.6
	RFreg	306.9	309.9	311.5	239.1
	XGBreg	3.2	238.9	4.1	229.9
	FFNN	690.3 (133.8^h^)	151.5 (130.0^h^)	723.3 (133.7^h^)	154.0 (129.8^h^)
	LSTM	1271.5	171.0	1330.0	174.2

^a^MAE: mean absolute error.

^b^MAEs for predicting the time of the next snack having high saturated fats, salt, or sugar in minutes using all features (fractional part of a minute is indicated as decimals). A lower MAE is better. The average across participants is provided.

^c^LR: linear regression.

^d^RFreg: random forest regressor.

^e^XGBreg: Extreme Gradient Boosting regressor.

^f^FFNN: feed forward neural network.

^g^LSTM: long short-term memory.

^h^The numbers in parentheses refer to model performance after applying regularization and early stopping.

For the Dutch dataset, errors were high. The FFNN model was the best performing model when using both 4 and 12 time bins. The training MAE for the FFNN model was much higher than the testing MAE, suggesting underfitting of data but good generalization. To assess whether the training MAE can be reduced, regularization and early stopping with the FFNN model were employed for the Dutch dataset.

The results can be explored in 2 ways. First, the scatter plots of residuals can be considered for the best performing version of each model. For most noisy processes, the default expectation is that residuals should be evenly distributed around zero, with the spread of the residuals dependent on the amount of noise and the quality of the model. A narrow spread of residuals around zero is generally indicative of good quality predictions [55]. Model residuals with scatter plots are presented in Multimedia Appendix 2. Overall, nonlinear models, particularly random forest, XGBoost, and FFNN, show the most potential for accurately predicting the time until the next HFSS snack, with the predominance of positive residuals being advantageous for just-in-time interventions (since positive residuals indicate that the prediction is earlier than the actual event).

Second, feature importance can be examined in the prediction task. Although there were only 3 features for prediction (time bin, location, and day of the week) and all of them appeared important, there might be enough information for prediction after eliminating day of the week or location. Feature importance analysis is one way to proceed in this case, but because of the approach used for model fit (individually for each participant), this was deemed impractical. Therefore, instead, we carried out 2 feature ablation analyses, one for location and another for day of the week. For the UK dataset, both these features appeared essential for the prediction task, since eliminating either one of them reduced model performance quite substantially (without either of the features, model performance became equivalent to that of the grand mean model) (Tables 3 and 4). On the other hand, for the Dutch dataset, the reduction in model performance in the ablation analyses was milder, indicating that prediction could be based on a subset of the available features.

**Table 3:** Location ablation analysis of the machine learning algorithms for predicting consumption of snacks with high saturated fats, salt, or sugar in the UK and Dutch datasets.

Dataset and model			Training set with 4 time bins (MAE^a,b^)		Testing set with 4 time bins (MAE^b^)		Training set with 12 time bins (MAE^b^)		Testing set with 12 time bins (MAE^b^)
**UK dataset**									
	Grand mean	29.2		47.3		29.2		47.1	
	LR^c^	17.1		>10,000^d^		13.8		>10,000^d^	
	RFreg^e^	15.3		52.2		12.1		42.5	
	XGBreg^f^	10.2		49.9		4.2		46.6	
	FFNN^g^	26.0		45.9		26.0		45.9	
	LSTM^h^	23.4		55.4		23.5		55.4	
**Dutch dataset**									
	Grand mean	1049.0		1397.3		1049.0		1398.0	
	LR	132.4		>10,000^d^		76.1		>10,000^d^	
	RFreg	370.4		267.6		341.6		276.3	
	XGBreg	73.4		222.5		23.1		258.8	
	FFNN	690.3		151.5		690.3		151.5	
	LSTM	1271.5		171.0		1271.5		171.0	

^a^MAE: mean absolute error.

^b^MAEs for predicting the time of the next snack having high saturated fats, salt, or sugar in minutes using all features (fractional part of a minute is indicated as decimals). A lower MAE is better. The average across participants is provided. Values are based on time bin and day of the week only.

^c^LR: linear regression.

^d^Extremely poor performance with a very high error.

^e^RFreg: random forest regressor.

^f^XGBreg: Extreme Gradient Boosting regressor.

^g^FFNN: feed forward neural network.

^h^LSTM: long short-term memory.

**Table 4:** Day of the week ablation analysis of the machine learning algorithms for predicting consumption of snacks with high saturated fats, salt, or sugar in the UK and Dutch datasets.

Dataset and model			Training set with 4 time bins (MAE^a,b^)		Testing set with 4 time bins (MAE^b^)		Training set with 12 time bins (MAE^b^)		Testing set with 12 time bins (MAE^b^)
**UK dataset**									
	Grand mean	29.2		47.3		29.2		47.1	
	LR^c^	21.1		>10,000^d^		15.2		>10,000^d^	
	RFreg^e^	21.1		45.9		16.9		39.9	
	XGBreg^f^	20.2		46.2		13.8		46.4	
	FFNN^g^	26.0		45.8		26.0		45.9	
	LSTM^h^	23.5		55.4		23.5		55.5	
**Dutch dataset**									
	Grand mean	1049.0		1397.3		1049.0		1398.0	
	LR	152.7		>10,000^d^		96.2		>10,000^d^	
	RFreg	416.0		284.4		360.9		229.3	
	XGBreg	135.3		212.2		73.5		197.6	
	FFNN	690.3		151.5		690.3		151.5	
	LSTM	1271.5		171.0		1271.5		171.0	

^a^MAE: mean absolute error.

^b^MAEs for predicting the time of the next snack having high saturated fats, salt, or sugar in minutes using all features (fractional part of a minute is indicated as decimals). A lower MAE is better. The average across participants is provided. Values are based on time bin and location only.

^c^LR: linear regression.

^d^Extremely poor performance with a very high error.

^e^RFreg: random forest regressor.

^f^XGBreg: Extreme Gradient Boosting regressor.

^g^FFNN: feed forward neural network.

^h^LSTM: long short-term memory.

An alternative, exploratory analysis is presented in Multimedia Appendix 3, which illustrates a different way to use the present data. This involved a sensitivity/specificity analysis, and the focus was to predict unhealthy snacks within windows of a certain size. This analysis was arguably better suited to the specific goal of building JITAIs. It was framed as a timing analysis for the delivery of a hypothetical intervention, which involved testing whether predicted snacking times fell within time windows of 120, 60, and 30 minutes before the actual HFSS snack time. This analysis was carried out only for the FFNN model, because FFNN residuals were notably positive and thus indicated early predictions, which is a desirable property for JITAIs. We briefly summarize the results from this analysis on individual behavior. Assuming the delivery of an intervention 45 minutes before a predicted snack, 90% of predictions would occur within a 1-hour window before the actual snack for the UK dataset and the Dutch dataset. If the acceptable window between the intervention and snack is reduced to 30 minutes (and the intervention is delivered 20 minutes before the prediction), the accuracy would be 75% and 74% for the UK dataset and the Dutch dataset, respectively.

## Discussion

### Principal Findings

Interventions can be made more effective by delivering messages at the right time before the target behavior is performed, as is the case with JITAIs (eg, [17,18]). ML can in principle help. The goal of this study was to design an app to collect data on HFSS snacking behavior in order to explore the capacity of well-known ML methods to capture the underlying statistical structure.

Over a period of 4 weeks, 111 participants provided data on HFSS snacking, using our Snack Tracker app, and this was considered the UK dataset. In addition, we analyzed an analogous dataset from the Netherlands (100 participants with a varying weight status), which was considered the Dutch dataset. In both cases, a number of preprocessing steps were carried out. We considered dividing a regular day into 4 and 12 time bins. We explored 4 established ML models, namely RFreg, XGBreg, FFNN, and LSTM, in the 2 datasets for predicting the number of minutes to the next HFSS snack (given the time bin in which HFSS snacking occurred) in each participant separately. The models were able to predict the time until the next HFSS snack with reasonable accuracy (the MAE was low at about 17 minutes in the UK dataset and was 130 minutes in the Dutch dataset). Although errors of 130 minutes (more than 2 hours) may appear large, this finding should be evaluated in the context of behaviors that themselves might occur only once every several hours. In many cases, prediction on the basis of the grand mean was inferior, demonstrating that the models were able to extract some regularity from the data. The residual analysis revealed reasonably narrow distributions, with desirable (in most cases) properties, such as positive residuals. Positive residuals mean that the prediction of the next HFSS snack is before the actual event, making them preferable (compared to negative residuals) for JITAIs. It would have been nice to offer a simple summary conclusion regarding which model performed better. Although there was some evidence that the FFNN model was overall superior, this was based mostly on the Dutch dataset. It makes sense to be cautious about overall model adequacy, and more analyses like the present ones are needed.

### Strengths

A strength of the study is the use of 2 datasets involving different populations, sampling approaches, and data collection methods. However, there is concern regarding the difference in MAE values between the UK and Dutch datasets. This may be attributed to differences in participant selection criteria between the 2 datasets. In the UK dataset, participants were selected based on their reported frequency of unhealthy snacking, and specifically, those who consumed two or more unhealthy snacks daily were considered. This likely led to a more homogeneous group in terms of snacking behavior, contributing to the lower MAE values. On the other hand, the Dutch dataset had a broader participant base without prior assessment of the snacking habit. The Dutch dataset included both people with a healthy weight and those who were overweight, but the UK dataset did not select participants based on BMI and instead included participants who consumed two or more unhealthy snacks daily. There were also differences in methodology and sampling techniques, which might have led to differences in the variability of snacking behavior and thus the capacity of the ML algorithms to perform well. Another point concerns the difference in the cultural context and how this might impact snacking behavior. For example, people in the United Kingdom (vs those in the Netherlands) eat less fruit (295 g vs 461 g per day; Food and Agriculture Organization of the United Nations, 2023) and are more likely to experience obesity (30% vs 23% of adults) [56].

Overall, there were several potential differences between the Dutch and UK datasets, and examining the performance of the ML algorithms separately for each dataset was an interesting exercise. Conversely, considering the range of differences mentioned above, there was little justification for combining the 2 datasets into a single analysis.

A major difference between the UK and Dutch datasets was related to the dependence on the additional features of location and day of the week over and above the main prediction feature of time bin. While prediction dropped substantially after eliminating either of these 2 additional features in the UK dataset, this was not the case in the Dutch dataset (prediction was equivalent with and without the 2 features). It is possible that location coding in the Dutch dataset was less accurate than that in the UK dataset. In the UK dataset, we specifically asked for location in terms of home, work, or other, which corresponded exactly to the feature coding in the analyses, but in the Dutch dataset, the location variable had to be derived from other data. However, this is a fairly weak reason (there is reasonable confidence in how the location variable was coded in the Dutch dataset), and in any case, this argument does not apply to the day of the week feature. More generally, there might have been more variation in the contextual cues that drive HFSS behavior between the 2 countries. At a preliminary level, it could be argued that the food environments between the 2 countries are broadly similar, but clearly, this is a possibility that cannot be addressed without further work.

From a technical point of view, a feature of the present approach is that the choice of methods represents relevant ML models for this type and size of dataset, without an attempt to develop a customized approach. Our emphasis was on how good prediction can be with standard ML algorithms. It is therefore interesting that the selected models mostly outperformed the baseline grand mean model. Models based on decision trees (XGBoost and random forest) performed competitively in predicting the next HFSS snack, but overall, neural networks (FFNN and LSTM) performed somewhat better. This finding may change with an increase in the number of data points, as neural networks, particularly deep ones, benefit greatly from large datasets. Moreover, there are alternative neural network models that merit examination, such as the Gated Recurrent Unit network, which is fast, requires little memory, and sometimes has better performance than LSTM [57]. One direction for future work is to determine how to use the characteristics of the present modeling challenge (such as sparsity) to fine-tune learning algorithms.

### Limitations

There are several technical challenges that need to be mentioned. First, behaviorally, for many users, it is difficult to commit to providing data for a long time, before interesting predictions are made. Even if most information is passively obtained, there would still be a small burden to record snacks. Therefore, it is worth exploring online ML, whereby data streams are used to continuously update a model. Another approach is to entirely obviate the need for user input through the use of sensor data combined with ML (digital phenotyping work mentioned earlier, eg, [30,31]). For example, Bangamuarachchi et al [58] reported some promising results concerning the identification of eating and noneating events, using only passive smartphone data (also see the study by Chen et al [59]). An important future direction would be to compare the quality of prediction from data collected actively versus passively, though the 2 approaches should not necessarily be seen in competition (eg, sensor data could be employed to validate active data entry).

The issue of recording snacks has another facet in terms of the change potential stemming from awareness of a behavior [11]. While this is a desirable effect of data collection (and indeed consistent with other research, eg, research in alcohol abuse; see the study by Pimpini et al [11]), it is a complicating factor regarding the validity of the dataset in that observation of the behavior impacts the behavior itself. However, it is very difficult, if not impossible, to disentangle such factors from the general variability in behaviors, such as HFSS snacking, which are inherently stochastic and subject to change from random environmental changes (eg, periods of stress or going on holiday). There is a profound challenge here, which goes beyond current standard practice in ML.

Second, a related challenge is that of data sparseness. ML models work better with more data. With a larger sample, it might be possible to examine whether there are natural groupings in HFSS snacking behavior, defined by, for example, demographic characteristics, personality traits, physiological differences, or eating behavior traits (eg, [60-63]). Different groups of this kind might display higher regularity in the HFSS eating behavior. Thus, categorizing a new participant against a pre-existing classification of individuals might inform our confidence of how well one can predict the behavior of that participant (eg, see Study II in the report by Spanakis et al [28]). Currently, the form of such a classification is unclear. Researchers could rely on ML to identify appropriate aspects and then attempt to interpret them against eating behavior characteristics.

Third, there is a question of whether the sample size was adequate. We have already mentioned the lack of established guidelines on sample size to achieve particular levels of sensitivity in ML work [46], as well as noted research similar to ours with similar sample sizes (eg, Spanakis et al [28]). Nonetheless, given the limitations in sampling, it can be questioned whether the present sample is sufficiently representative of the general population. Participants were recruited through Prolific Academic, which is one of the most widely employed crowd-sourcing platforms for behavioral experiments. Their age characteristics and gender balance revealed no peculiarities. The recruited participants were biased toward those who consume more HFSS snacks and those with enough technology familiarity to use our app. Nonetheless, they represent participants who would take part in future interventions, based on the kind of HFSS snacking prediction considered here.

Fourth, in this work, we only employed day of the week, time, and location (coarsely coded). In contrast, the studies by Arend et al [25], Forman et al [26], and Spanakis et al [28] are all sophisticated examples of similar predictive modeling but based on more information. The use of a greater number of features has the potential for more accurate prediction. Regarding HFSS snacks, the times of meals and drinks are particularly pertinent. However, we wanted to explore the predictive potential based on information that would be as straightforward as possible for participants to provide. The motivation for such an approach is that any scheme suitable for roll-out to the general population would benefit from being as unobtrusive as possible in terms of requests for information. In our app, location required participant input, but in future iterations, this feature could be automated through simple look-up tables involving geolocation data and the prerecorded locations of particular users for work, home, etc. Additional information that could be recorded automatically include sensor data from sophisticated smartwatches, such as heart rate, blood pressure, and possibly blood sugar, or whether the individual is alone or with company. There could be a tradeoff here among predictive power, usability, and participant fatigue, which will require more work before clarification.

A related point is whether an autoregressive approach might be more fruitful. Our analyses were based on taking all time bins in which there were snacks and trying to predict the time (minutes) to the next snack (given the time bin, location, and day of the week). What if *all* time bins are considered as input for the models, regardless of whether there is a snack, and the presence of a snack is used as an additional feature? In brief, such an approach does not work with the present data. The likely problem is the sparseness of snacking bins relative to no-snacking bins, and thus, the dataset is skewed toward the latter. This kind of data skew is a recognized problem in ML, leading to poor performance of ML models [64]. Such problems from undersampling of snacking instances might be mitigated in larger datasets.

Fifth, the possibility of using more extensive sensor data goes hand in hand with the intriguing suggestion to individualize the ML approach [65]. There are merits and demerits for both collecting more data per participant (to allow, for example, individualization) and adopting a more general approach with minimal data collection per participant (Spanakis et al [28] provide a discussion of the tradeoff between individualization and generality). It is worth noting, however, that even with the present minimal data collection and a general approach to ML, it was possible to achieve reasonably low prediction errors.

Sixth, HFSS snacking behavior is not necessarily stable and might vary depending on factors such as the time of the year. Accordingly, an ML approach would need to be able to adapt to any changes in behavior. The difficulty then is how to distinguish between routine deviations in baseline behavior and changes in the behavior itself. Online ML might again offer promise in addressing such a challenge [66]. These considerations also challenge a perception that perhaps it would be more appropriate to base predictions on stationary data.

Challenges like those mentioned above are not independent of the goal of the prediction. Arguably, if the purpose of prediction is to carry out an intervention, it may be valuable to know not only the time of an HFSS snacking event, but also additional variables, such as whether particular emotions preceded a snacking event. Currently, for the reasons outlined above, it seems unlikely that a practical method (ie, beyond research) based on multiple variables or EMAs can be developed, but future personal electronic devices may make the collection of the data possible. Participants are likely to engage with intensive data-collection procedures if they are sufficiently motivated to accomplish goals related to dietary change, weight reduction, etc.

### Conclusions

The examination of standard ML algorithms produced promising findings concerning the prediction of HFSS snacking based on sparse data involving only previous occasions of such snacking behavior. There are promising behavioral implications given that a reasonable degree of predictive accuracy was possible even with limited data (in terms of both features and the window for data collection). One direction for application concerns using prediction data to deliver behavior change techniques for HFSS snacking behavior (eg, [16,67]). These behavior change techniques could be delivered in several different ways (eg, by text or audio messages) to increase engagement and could also be selected and personalized for the individual. Additionally, the ML work by itself could be applied to other behaviors targeted for reduction, such as smoking and alcohol consumption.

The present results offer a foundation for further exploring how ML methods can be used in health psychology and provide directions for further research, which can be both more technically oriented and focused on behavioral applications and extensions.

## Appendix

Multimedia Appendix 1 Additional information on the study methods.

Multimedia Appendix 2 Model residuals concerning the objective of predicting the time until the next unhealthy snack.

Multimedia Appendix 3 An alternative predictive objective.

## Abbreviations

API: application programming interface
EMA: ecological momentary assessment
FFNN: feed forward neural network
HFSS: high saturated fats, salt, or sugar
JITAI: just-in-time adaptive intervention
LSTM: long short-term memory
MAE: mean absolute error
ML: machine learning
RFreg: random forest regressor
XGBreg: Extreme Gradient Boosting regressor

## Data Availability

The data and code in this study are publicly available through an Open Science Foundation page [68].
