# Supplementary material for: Prediction of Snacking Behavior Involving Snacks Having High Levels of Saturated Fats, Salt, or Sugar Using Only Information on Previous Instances of Snacking: Survey- and App-Based Study
Source: JMIR Med Inform. 2025 Apr 23;13:e57530. doi: 10.2196/57530 (PMC12059507; doi:10.2196/57530)
Supplement: Multimedia Appendix 1 [file medinform_v13i1e57530_app1.doc]

**Multimedia Appendix 1.** Additional information on the study methods.

The questions below were given to participants during the first, screening part of the study.

Table S1. Demographics questions in the first part of the data collection procedure.

| **Question** | **Cardinality** | **Choices\value** |
| --- | --- | --- |
| What is your age? | - | 18-60 Male, Female, Other White Mixed/Multiple ethnic groups Asian/Asian British Black/African/Caribbean/Black British Other ethnic group Unknown Prefer not to say |
| What is your gender? | 3 | In paid employment Studying full time Unemployed Homemaker Retired |
| Please indicate your ethnicity: | 7 | Other (please specify) |
| Please indicate your employment status: | 5 | 18-60 Male, Female, Other White Mixed/Multiple ethnic groups Asian/Asian British Black/African/Caribbean/Black British Other ethnic group Unknown Prefer not to say |
| What is your weight? | - | In paid employment Studying full time Unemployed Homemaker Retired |
| What is your height? | - | Other (please specify) |

Table S2. Questions regarding motivation for healthy eating in the survey

| **Question** | **Cardinality** | **Choices\value** |
| --- | --- | --- |
| 1. On a typical day, approximately how - many sugary, salty or fatty snacks do you usually consume in between main meals? | - | - |
| 2. Are you currently dieting to lose 3 weight? (dieting) | 3 | Yes , No, I’d rather not say |
| 3. It is important to me to watch my weight (IWW) | 7 | 1-7 scale anchored by  ‘strongly disagree’ and ‘strongly agree’ |
| 4. It is important to me to eat a healthy diet (IEHD) | 7 | 1-7 scale anchored by ‘strongly disagree’ and ‘strongly agree’ |

NB. Questions 2 and 3 were Likert scales, with a number of responses as indicated.

| 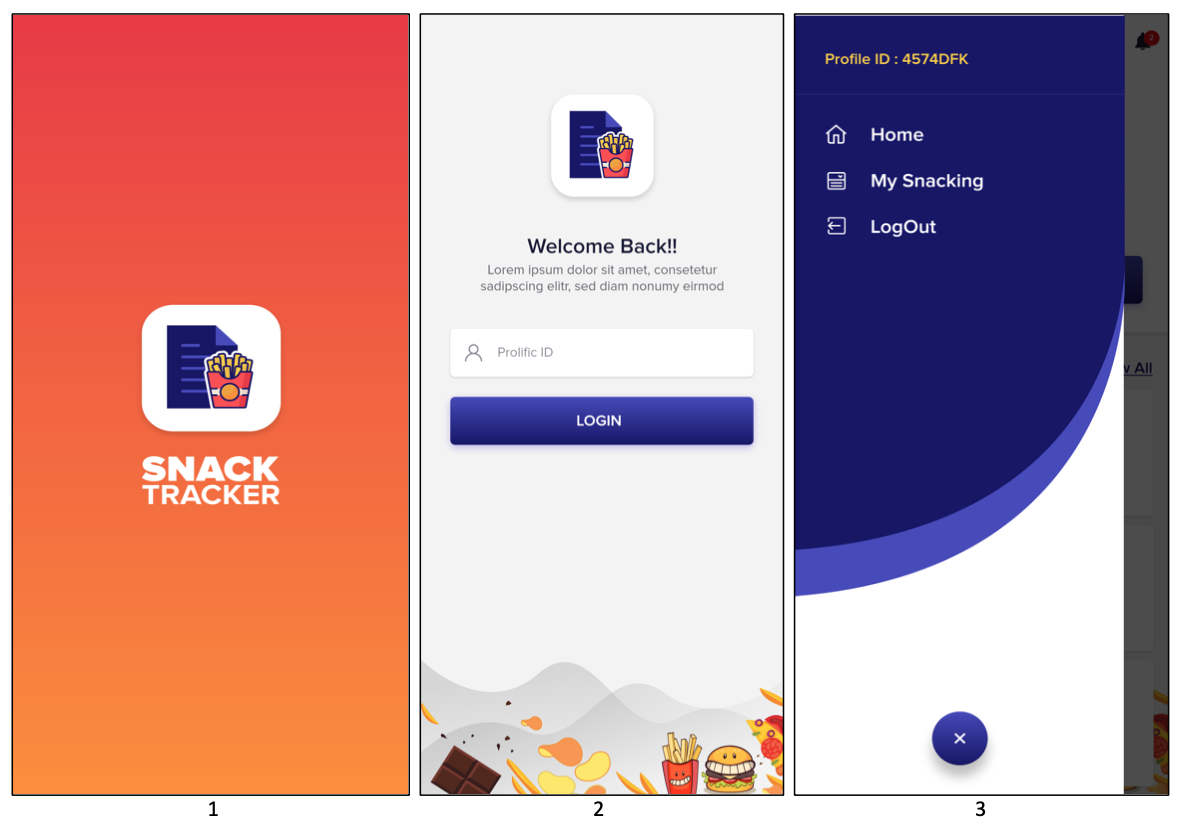 |
| --- |
| 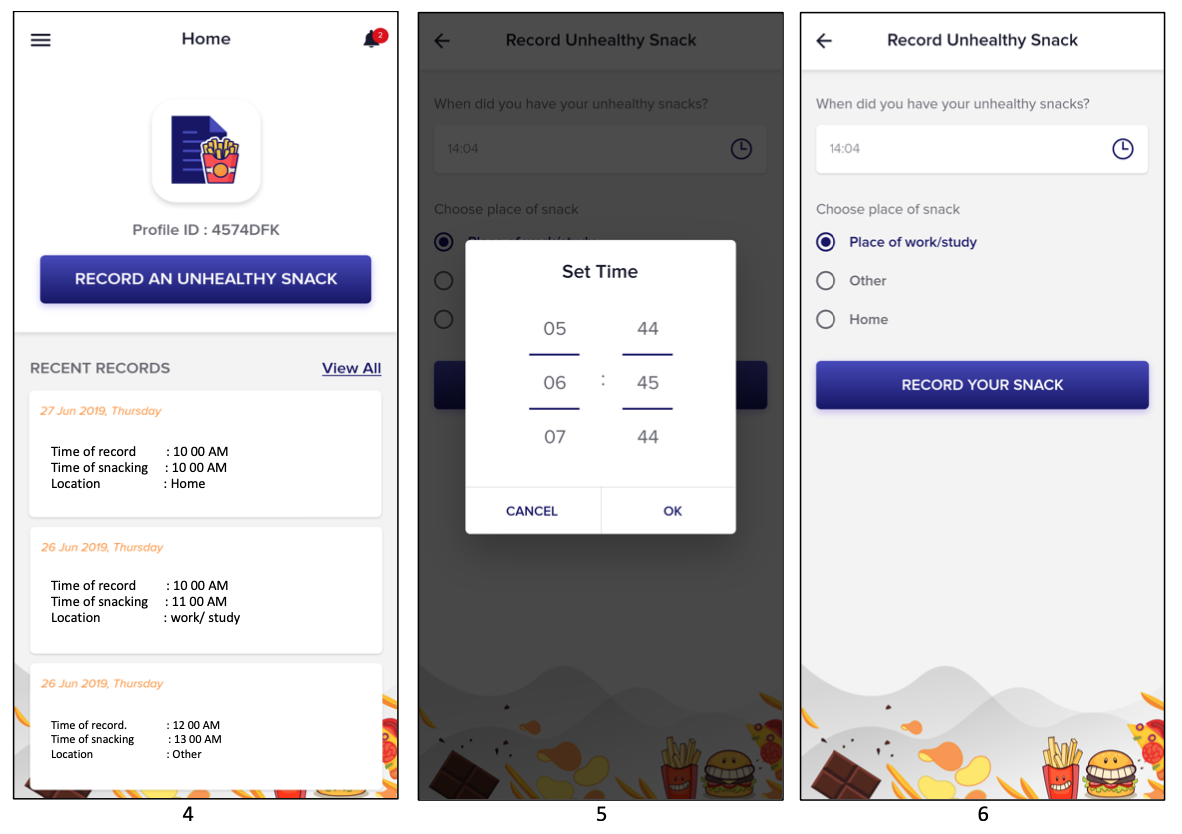 |
| 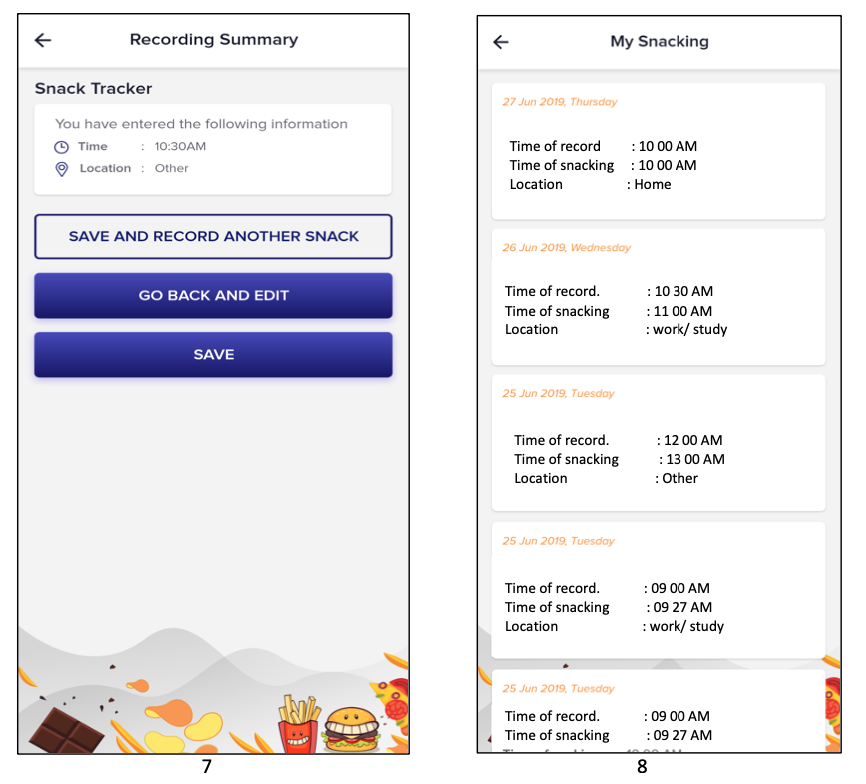 |

Figure S1. (larger version of Figure 3). The frontend of the Snack Tracker app: 1, splash screen; 2, login screen; 3, home screen; 4, new snack screen; 5, time recording screen (time picker); 6, location recording screen; 7, recording save screen; 8, review recordings summary.

Full instructions concerning how the idea of HFSS snacks was explained to participants.

In the invitation message to participate in the second part of the study, we informed participants about the specific task and how to record their snacking behavior. The relevant part of the message that was employed in the invitation is as follows:

“Thank you for completing the first part of our study. We would like to invite you to take part in the second part! The second part takes place over a period of 28 days. All you have to do is to simply record every instance of snacking behavior. We are interested in snacks high in sugar, salt, or fat, which includes:

*Sugary snacks: biscuits, cake, chocolate, sweets, sugary desserts, sugary breakfast cereals, sugary granola bars, sodas, milkshakes, flavoured milk drinks.

*Salty and fatty snacks: crisps, salted nuts, salted popcorn, pretzels, chips, burgers, cheese.

We are using the word ‘snack’ to refer to any food that is eaten in between main meals. Every time you have a snack high in sugar, salt, or fat, you will have to use an App that will be provided to you, just record the location (work/ study place, home, or other) and the time of the snack – and that’s it! Recording a snack should take only a few seconds. You will be paid £16 on completion.

…”

Additional information regarding checks concerning participant engagement with the study

After approximately 15 days of their participation in the second part of the study, we sent a message to participants to (a) Check the average number of recordings per day to ensure data completeness (b) Verify if participants were receiving daily SMS reminders to stay on track.

In addition, some participants received a message if we noticed any anomalies, such as days with no recordings or a significant drop in the number of recorded snacks. These steps were taken to maintain data accuracy and consistency throughout the study and to provide participants with an opportunity to address any issues or discrepancies that might have arisen during the data collection process.
