# Supplementary material for: Prediction of Snacking Behavior Involving Snacks Having High Levels of Saturated Fats, Salt, or Sugar Using Only Information on Previous Instances of Snacking: Survey- and App-Based Study
Source: JMIR Med Inform. 2025 Apr 23;13:e57530. doi: 10.2196/57530 (PMC12059507; doi:10.2196/57530)
Supplement: Multimedia Appendix 2 [file medinform_v13i1e57530_app2.docx]

**Multimedia Appendix 2.** Model residuals concerning the objective of predicting the time until the next unhealthy snack.

We show residuals scatter plots of all considered models, for predicting the time until the next unhealthy snack. In Figures S1-S4, the residuals are calculated as true values minus predicted, so that positive residuals indicate that the prediction is earlier than the observed time and negative residuals indicate that the predicted time is later than observed. Figure S1 shows the residuals scatters for various models (Grand Mean, Linear Regression, RFreg, XGBreg, FFNN, and LSTM) applied to the UK dataset with 4 time bins. Figure S2 illustrates the residuals for the same models applied to the UK dataset with 12 time bins. Figures S3 and S4 display the residuals for these models applied to the Dutch dataset with 4 and 12 time bins, respectively.

As seen from the plots, the residuals from the Grand Mean model are widely scattered across all the datasets and time bins, indicating very poor prediction accuracy. Therefore, this model does not capture the variability of the data quite effectively. Similarly, Linear Regression (LR) residuals are also substantially scattered in both positive and negative values in all datasets and time bins, showing how the model is struggling with prediction accuracy.

RFreg residuals are closer to a normal distribution, with fewer outliers in all datasets and time bins, suggesting better and more consistent prediction accuracy compared to the Grand Mean and Linear Regression models. Similar to RFreg, XGBreg residuals are generally close to a normal distribution with few outliers, indicating good prediction accuracy. These models handle the variability in the data effectively across all datasets and time bins. However, as can be seen from the plots, the presence of some negative residuals is undesirable, as this indicates late predictions after the occurrence of the event.

The FFNN model mostly shows positive residuals, indicating earlier predictions across all datasets and time bins. While there are some large positive residuals, the model shows good accuracy. The LSTM model shows mainly positive residuals with very few negatives across all datasets and time bins. Although there are some extreme outliers, the FFNN and LSTM models generally provide earlier predictions, which can be advantageous for just-in-time interventions.

Overall, the residual analysis across the UK and Dutch datasets shows that non-linear models such as RFreg, XGBreg FFNN and LSTM perform better compared to Grand Mean and Linear Regression. As seen from the plots, RFreg and XGBreg have residuals closer to a normal distribution with fewer outliers, though some negative residuals indicate late predictions. FFNN and LSTM primarily show positive residuals, which is preferable to negative ones.


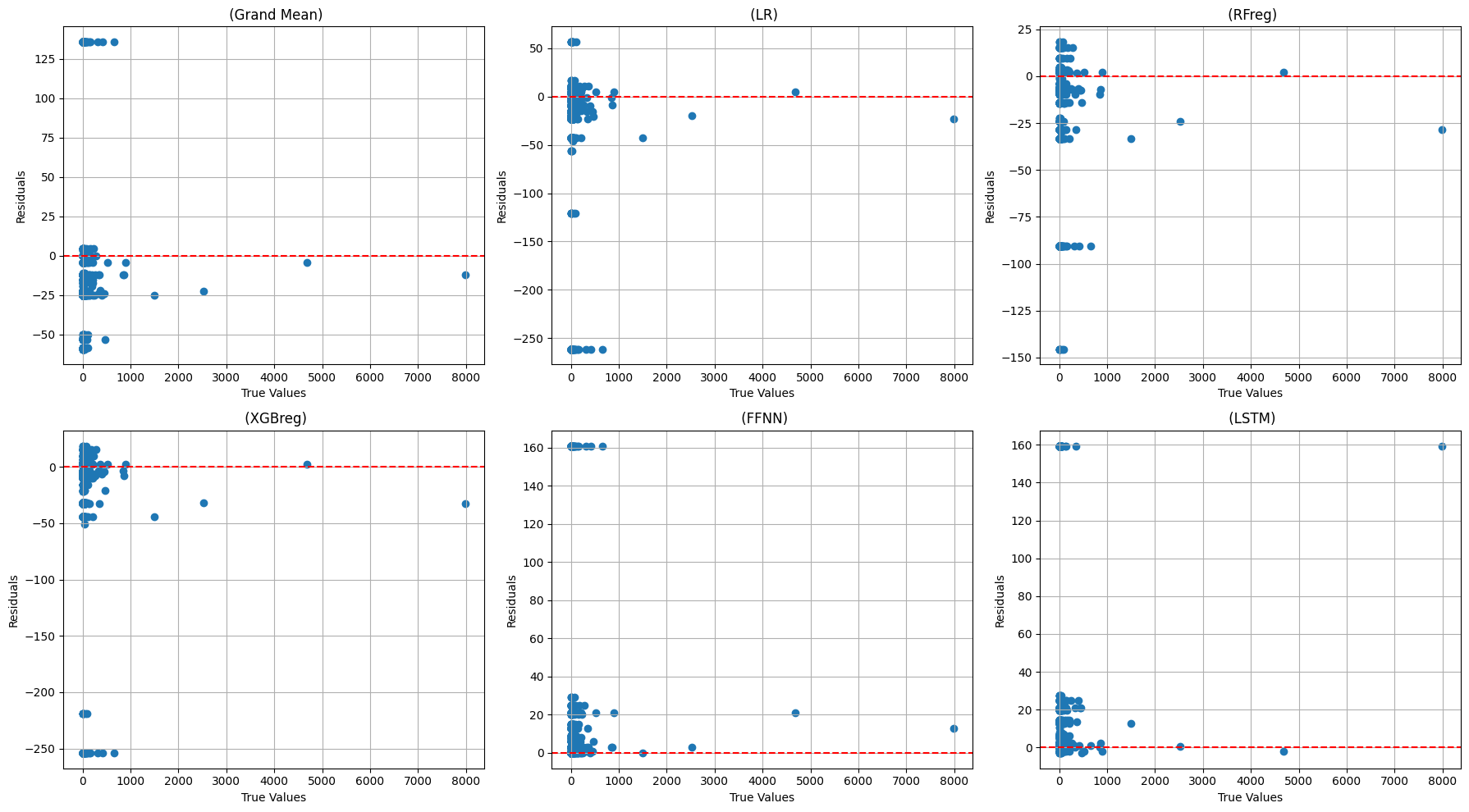


**Figure S1.** Scatter plot of residuals for all model predictions for the UK dataset with 4 time bins.


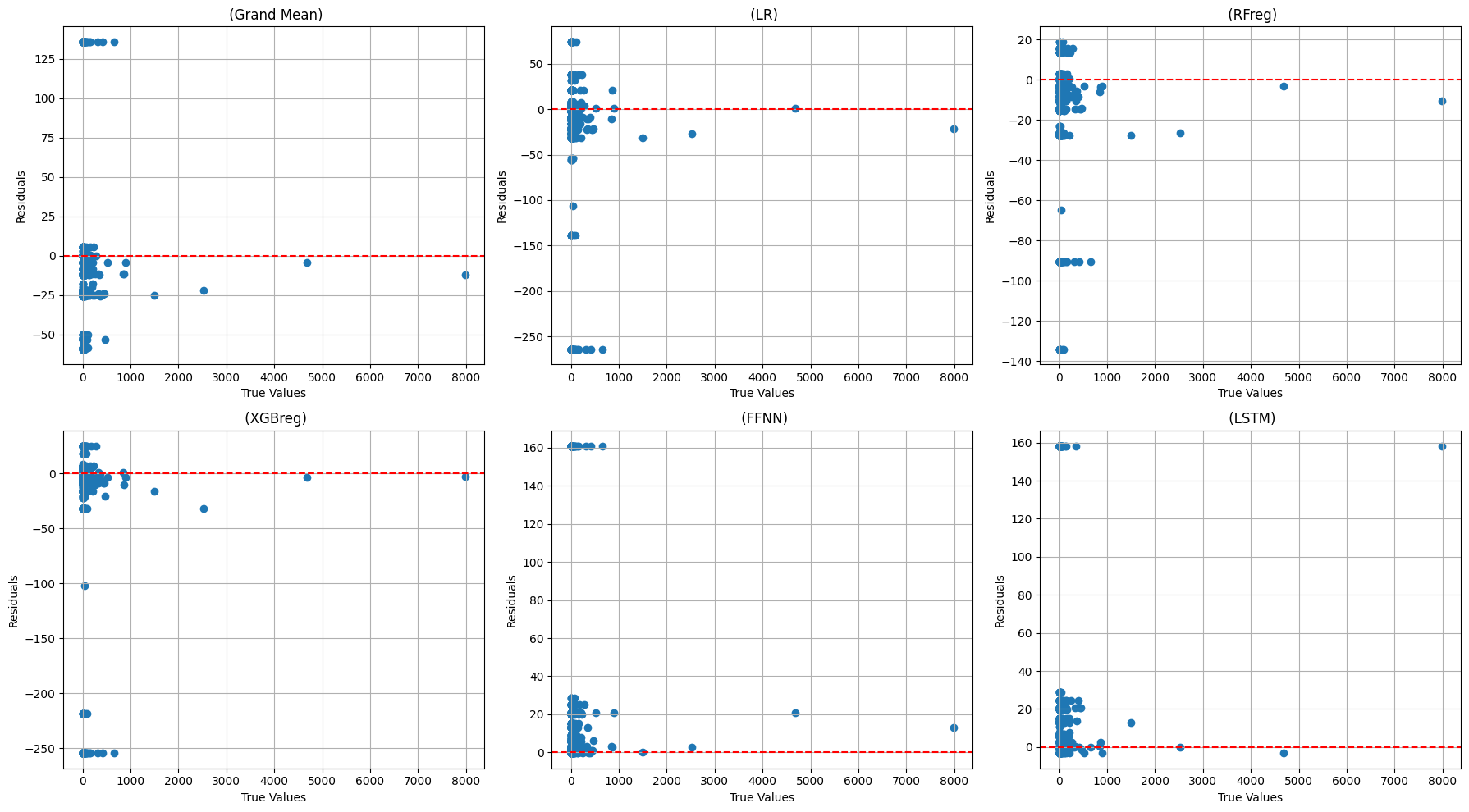


**Figure S2.** Scatter plot of residuals for all model predictions for the UK dataset with 12 time bins.


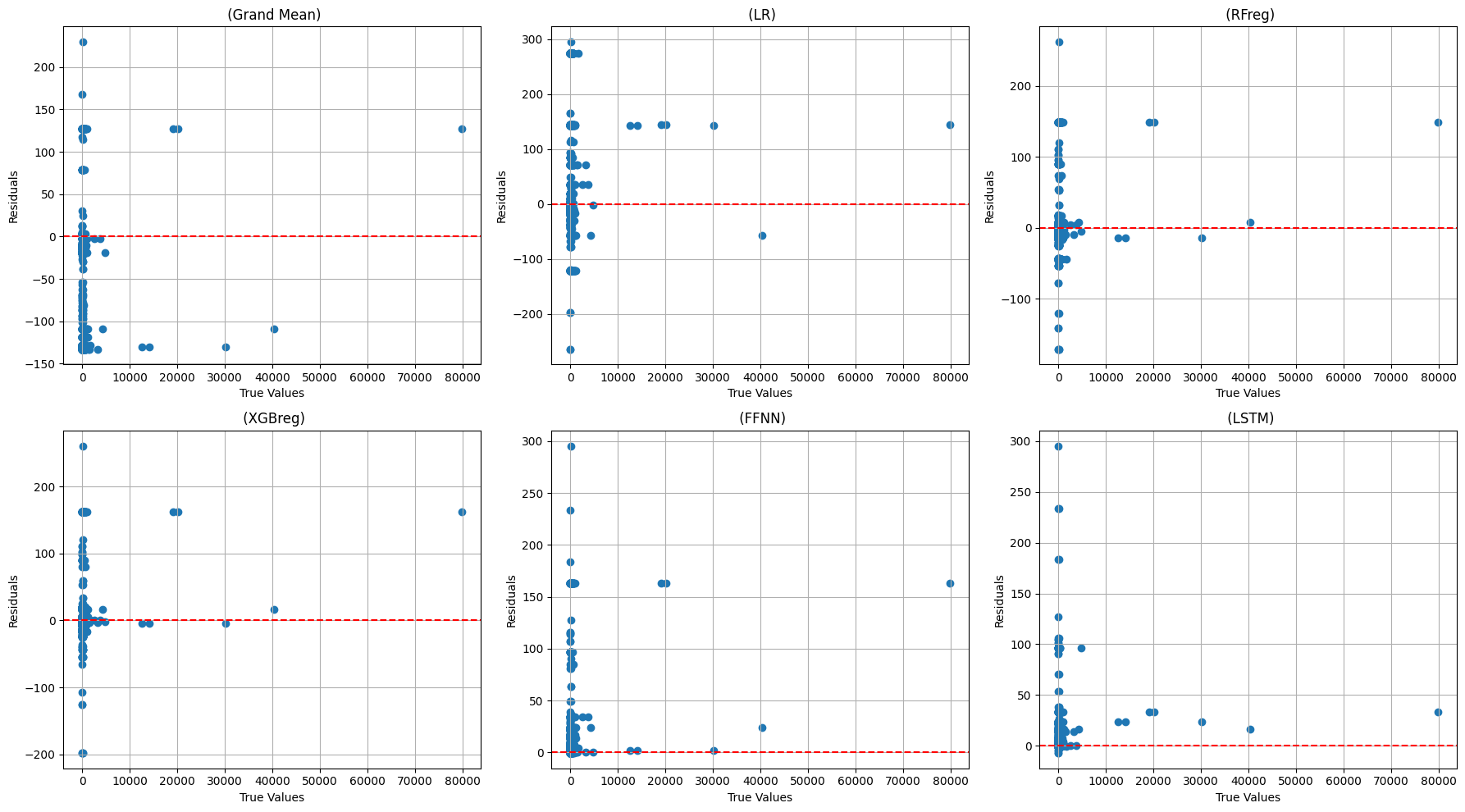


**Figure S3.** scatter plot of residuals for all model predictions for the Dutch dataset with 4 time bins.


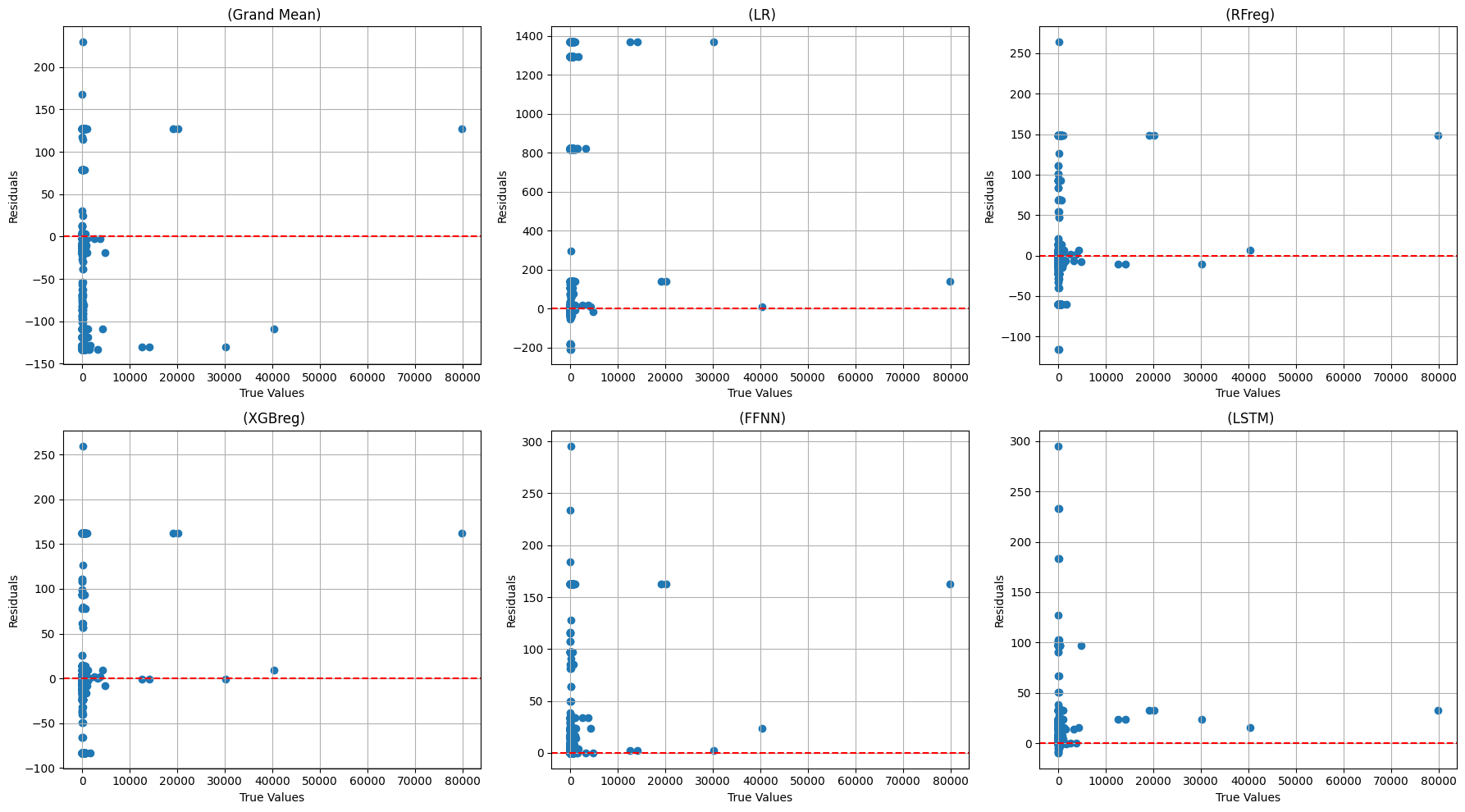


**Figure S4.** Scatter plot of residuals for all model predictions for the Dutch dataset with 12 time bins.
