# Supplementary material for: Prediction of Snacking Behavior Involving Snacks Having High Levels of Saturated Fats, Salt, or Sugar Using Only Information on Previous Instances of Snacking: Survey- and App-Based Study
Source: JMIR Med Inform. 2025 Apr 23;13:e57530. doi: 10.2196/57530 (PMC12059507; doi:10.2196/57530)
Supplement: Multimedia Appendix 3 [file medinform_v13i1e57530_app3.docx]

**Appendix 3**. An alternative predictive objective.

Health psychologists and related professionals might be interested in this work for the purpose of supporting behavior change with timed interventions. Here, we propose an analysis arguably more geared towards such applications, based on the speciﬁc timing criteria desirable for a putative behavior change intervention. That is, even though we do not employ an intervention, we offer an analysis concerning timings, as would be useful for constructing an intervention.

The logic of this analysis is as follows. An inﬂuential distinction is to understand thought as reﬂecting two types of processes, reﬂective and impulsive. We can consider human behavior change in terms of these processes. It is thought that unhealthy snacking may often be an automatic process [1,2]. Accordingly, many researchers agree on the importance of the delivery time of interventions (e.g., [3,4]). For example, the effectiveness of interventions has been demonstrated to vary depending on the timing of the delivery. Information we have been exposed to more recently tends to be more cognitively accessible. Nudges presented too early, relative to the targeted action, may be ineffective, because of forgetting, while nudges presented late may reduce the amount of time available for processing and executing a change [5]. In the context of predictive models, there is also the risk of the predicted time being later than the intervention, so that the intervention happens after the targeted event and the opportunity for change has been missed. In general, a nudge offered a short time before the behavior is desirable, but this has to be balanced against the risk of being too late.

Here, we evaluate the Feed forward Neural Network (FFNN) model’s performance in addressing the problem of predicting timing in unhealthy snacking behavior, by considering the distribution of residuals concerning the time for the next unhealthy snack and by producing the outcome with different timing parameters. That is, we want to consider the ability of FFNN to predict unhealthy snacks within windows of a certain size. Note, FFNN was the best model based on MAE and its residuals confirmed that it is a good candidate for hypothetical interventions due to its early predictions, as indicated by the mostly positive residuals.

We offer a sketch for the structure of a putative intervention, so as to make the prediction problem more precise. We define as Predicted Unhealthy Snacking Time (PST) the time until the next unhealthy snack. Then, we define the Proposed Intervention Time (PIT) by subtracting the Lead Time (LT) from PST, i.e., PIT = PST – LT. The idea is that LT would be used to control the timing of the intervention and determine how far in advance an intervention should be delivered. We use LT values of 10, 20, 30, 45, 60, 75, and 90 minutes. Different LT values would allow a researcher to determine the ideal balance between time precision and accuracy (in terms of percentage, see shortly), in relation to putative interventions for preventing unhealthy snacking.

We next define the Time Difference (TD) by subtracting the PIT from the Actual Unhealthy Snacking Time (AUT), i.e., TD = AUT- PIT. TD is the difference between the time at which the (putative) intervention was delivered and the time at which the person actually snacked. TD allows us to evaluate the precision of predictions (i.e., the PITs) and, specifically, whether they were within, before, or after time windows of differing widths, W, of 30, 60 and 120 minutes. For example, for W=30, we consider the intervention as ‘Hitting’ if snacking and intervention cooccur within a window of 30 minutes. Furthermore, we categorized PIT values as Early and Late accordingly. A hitting score can be defined as the percentage of instances where the hypothetical intervention's timing aligned with the actual unhealthy snacking occurrence – this is basically a measure of the accuracy of the intervention’s timing. For example, a hitting score of X% with LT=10 minutes suggests that the intervention was effective in being appropriately timed in X% of cases, assuming we aimed for the intervention to occur within 10 minutes prior to the unhealthy snack.

To summarize the relevant definitions:

PST: Predicted Unhealthy Snacking Time

LT: Lead Time (for an intervention to prevent an unhealthy snack, before the snack)

PIT = PST - LT: Proposed Intervention Time

AUT: Actual Unhealthy Snacking Time

TD = AUT – PIT: time difference (precision of predictions)

Each of the ﬁgures below shows the effectiveness of the hypothetical intervention, for different LT values, as well as Early, Hitting, or Late results, with respect to W values of 30, 60, and 120 minutes to the time window. We also separate results by time bin (4 and 12 time bins).

Figure A3.1 shows hitting results when W=30. Across different LT values, the highest hitting scores were obtained with LT=20 minutes. For the UK dataset with 4 time bins, the hitting score was 74%. For the UK dataset with 12 time bins, the hitting score was slightly higher at 75%. Similarly, for the Dutch dataset with both time bins (4 and 12), the hitting score was 75%. Figure A3.2 shows corresponding results with W=60. The most notable performance was 90% across all datasets and time bins with LT=45 minutes and in addition with LT=30 minutes for the UK dataset with 12 time bins. Considering W=120, in Figure A3.3, we observed remarkable success rates on both datasets and using 4 and 12 time bins. The best success rate was 90%, achieved with LT values of 45, 60, 75, and 90 minutes.

These results show that hitting scores did not vary considerably between the UK and Dutch datasets and across the two different ways to partition the day. The performance did not vary much based on different LT values, showing relatively stable results across the tested LT values. These results suggest that if we are aiming for a 30-minute window, it is better to use an LT of 20 minutes. A broadly analogous point applies to whether the analysis is carried out against data organized with 12 or 4 time bins. Overall, hitting scores were slightly higher with 12 time bins in a few cases (e. g. UK dataset within W=30 and W=60). Additionally, hitting scores were quite similar for both datasets across different time windows (W).

In summary, the shortest window time we investigated was 30 minutes. For this time window, we think that the modeling results show reasonable accuracy for hypothetical interventions, of 74% or 75% for the UK and Dutch datasets. In pilot results not reported here, shorter time windows (e.g., 20 or 15 minutes), were associated for performance too poor to be useful. Note, qualifications such as ‘good enough’, ‘useful’ etc. are offered as very preliminary: it is a difficult empirical challenge to establish what is a sufficient percentage of hits before an intervention is effective. Analogously, it is unclear what is the effective range between intervention and targeted behavior, a question further complicated by the fact that a behavior such as unhealthy snacking would be very noisy by itself. Finally, the choice of FFNN for this analysis was reasonable, but it has to be remembered that, in our current data, machine learning models performed to some extent equivalently. We think that interesting model differences would be evident only with larger data collection exercises, perhaps over a period of months, rather than weeks.


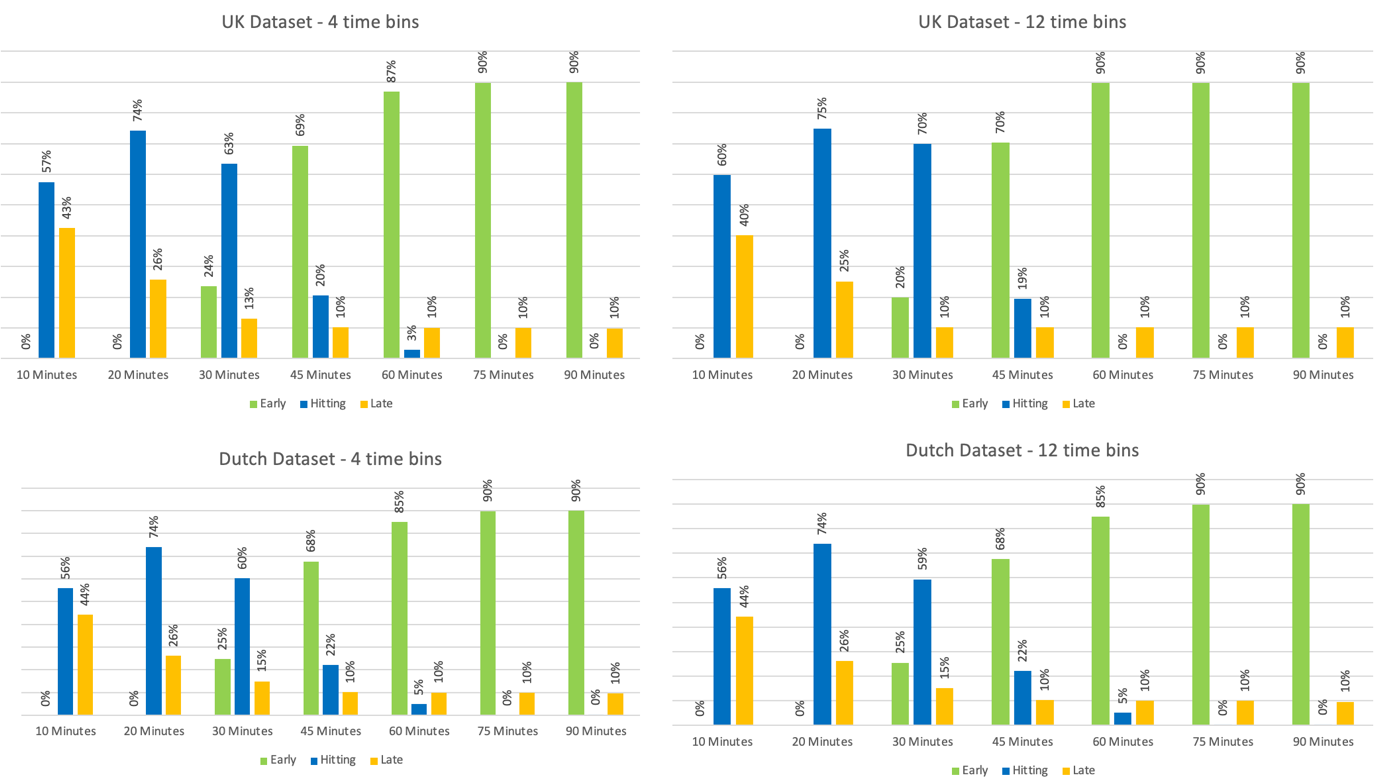


Figure A3.1. Early, hitting, and late results distribution for a 30-minute window for accepting an intervention, with 4 and 12 time bins, in the UK and Dutch datasets. The hitting score corresponds to the percentages in the ‘hitting’ column, in each case.


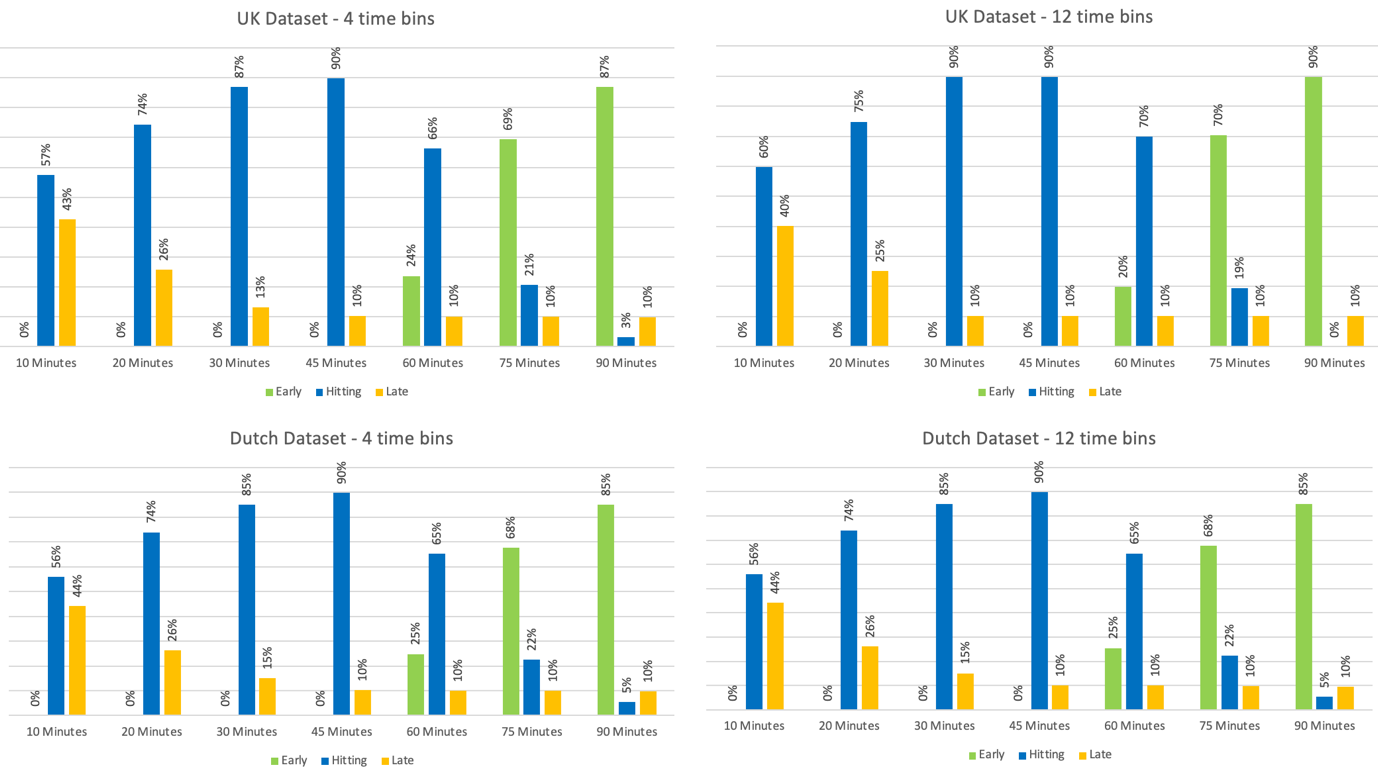


Figure A3.2. Early, hitting, and late results for a 60-minute window for accepting an intervention, with 4 and 12 time bins, in the UK and Dutch datasets. The hitting score corresponds to the percentages in the ‘hitting’ column, in each case.


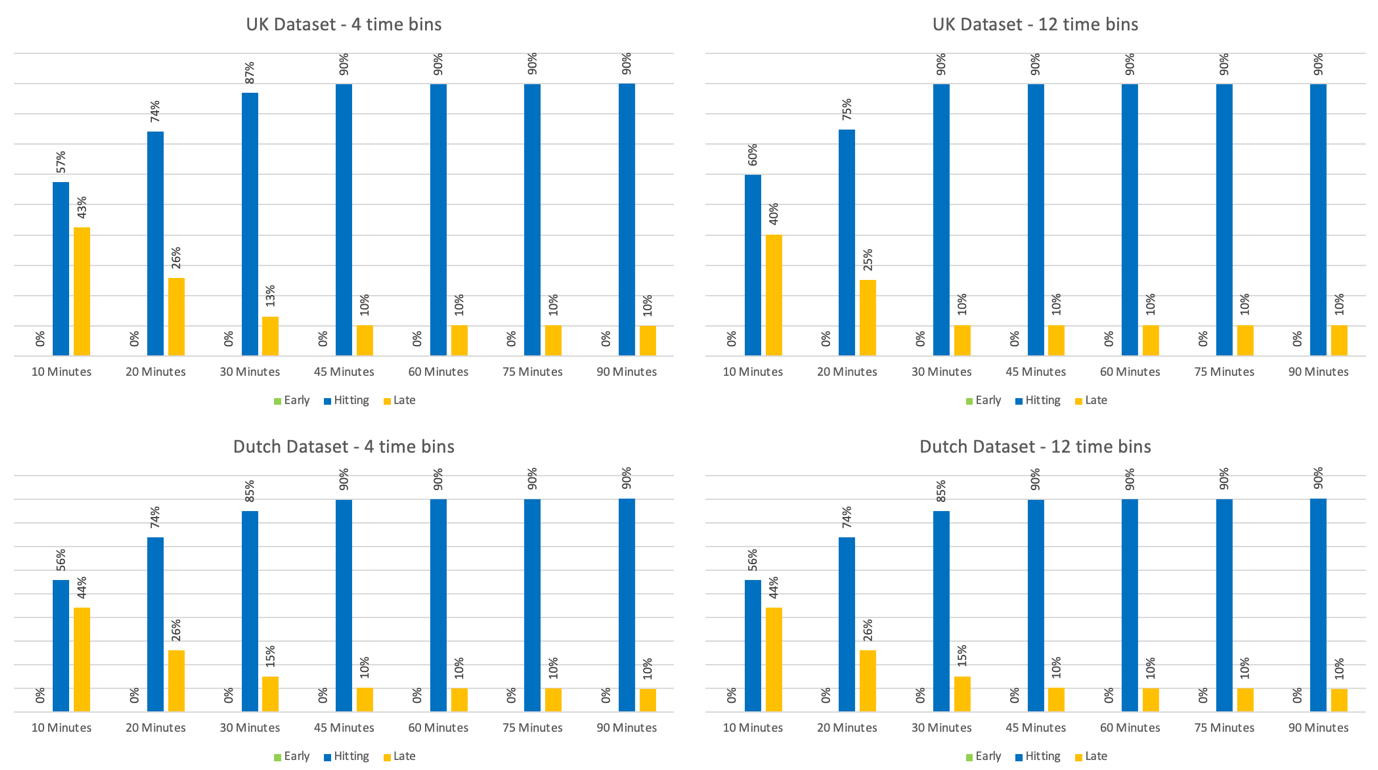


Figure A3.3. Early, hitting, and late results for a 120-minute window for accepting an intervention, with 4 and 12 time bins, in the UK and Dutch datasets. The hitting score corresponds to the percentages in the ‘hitting’ column, in each case.

Additional references

Neal, D. T., Wood, W., Wu, M., & Kurlander, D. (2011). The pull of the past: When do habits persist despite conflict with motives?. Personality and social psychology bulletin, 37(11), 1428-1437.

Verhoeven, A. A., Adriaanse, M. A., Evers, C., & de Ridder, D. T. (2012). The power of habits: Unhealthy snacking behaviour is primarily predicted by habit strength. British Journal of Health Psychology, 17(4), 758-770.

Hardeman, W., Houghton, J., Lane, K., Jones, A., & Naughton, F. (2019). A systematic review of just-in-time adaptive interventions (JITAIs) to promote physical activity. International Journal of Behavioral Nutrition and Physical Activity, 16, 1-21.

Intille SS, Kukla C, Farzanfar R, Bakr W. Just-in-time technology to encourage incremental, dietary behavior change. AMIA Annu Symp Proc 2003;2003:874.

Gillitzer C, Sinning M. Nudging businesses to pay their taxes: Does timing matter? J Econ Behav Organ 2020;169. doi: 10.1016/j.jebo.2019.11.020

Katy Tapper. Health Psychology and Behaviour Change: From Science to Practice. 1st ed. London, UK: Bloomsbury Academic; 2021. ISBN:9781350312340
